# Supplementary material for: Hydrophobic mismatch drives self-organization of designer proteins into synthetic membranes
Source: Nat Commun. 2024 Apr 11;15:3162. doi: 10.1038/s41467-024-47163-1 (PMC11009411; doi:10.1038/s41467-024-47163-1)
Supplement: Supplementary file 1 — Supplementary Information [file 41467_2024_47163_MOESM1_ESM.pdf]

Supplementary Information

Designed proteins self-organize into synthetic membranes via hydrophobic mismatch

Justin A. Peruzzi[1,2], Jan Steinkühler[2,3], Timothy Q. Vu[2,3], Taylor F. Gunnels[2,3], Vivian T. Hu[2,3], Peilong Lu[4,5,6], David Baker[7,8,9], Neha P. Kamat[2,3,10*]

[1]Department of Chemical and Biological Engineering, Northwestern University, Evanston, IL 60208, USA.

[2]Center for Synthetic Biology, Northwestern University, Evanston, IL, 60208, USA.

[3]Department of Biomedical Engineering, Northwestern University, Evanston, IL, 60208, USA.

[4]Key Laboratory of Structural Biology of Zhejiang Province, School of Life Sciences, Westlake University, Hangzhou, Zhejiang, China.

[5]Westlake Laboratory of Life Sciences and Biomedicine, Hangzhou, Zhejiang, China.

[6]Institute of Biology, Westlake Institute for Advanced Study, Hangzhou, Zhejiang, China.

[7]Department of Biochemistry, University of Washington, Seattle, WA 98195, USA.

[8]Institute for Protein Design, University of Washington, Seattle, WA 98195, USA.

[9]Howard Hughes Medical Institute, University of Washington, Seattle, WA 98195, USA.

[10]Chemistry of Life Processes Institute, Northwestern University, Evanston, IL 60208, USA.

*Corresponding author. Email: nkamat@northwestern.edu

Contents:

Supplementary Figures 1 - 17

Supplementary Table 1- 3

Supplemental Figures

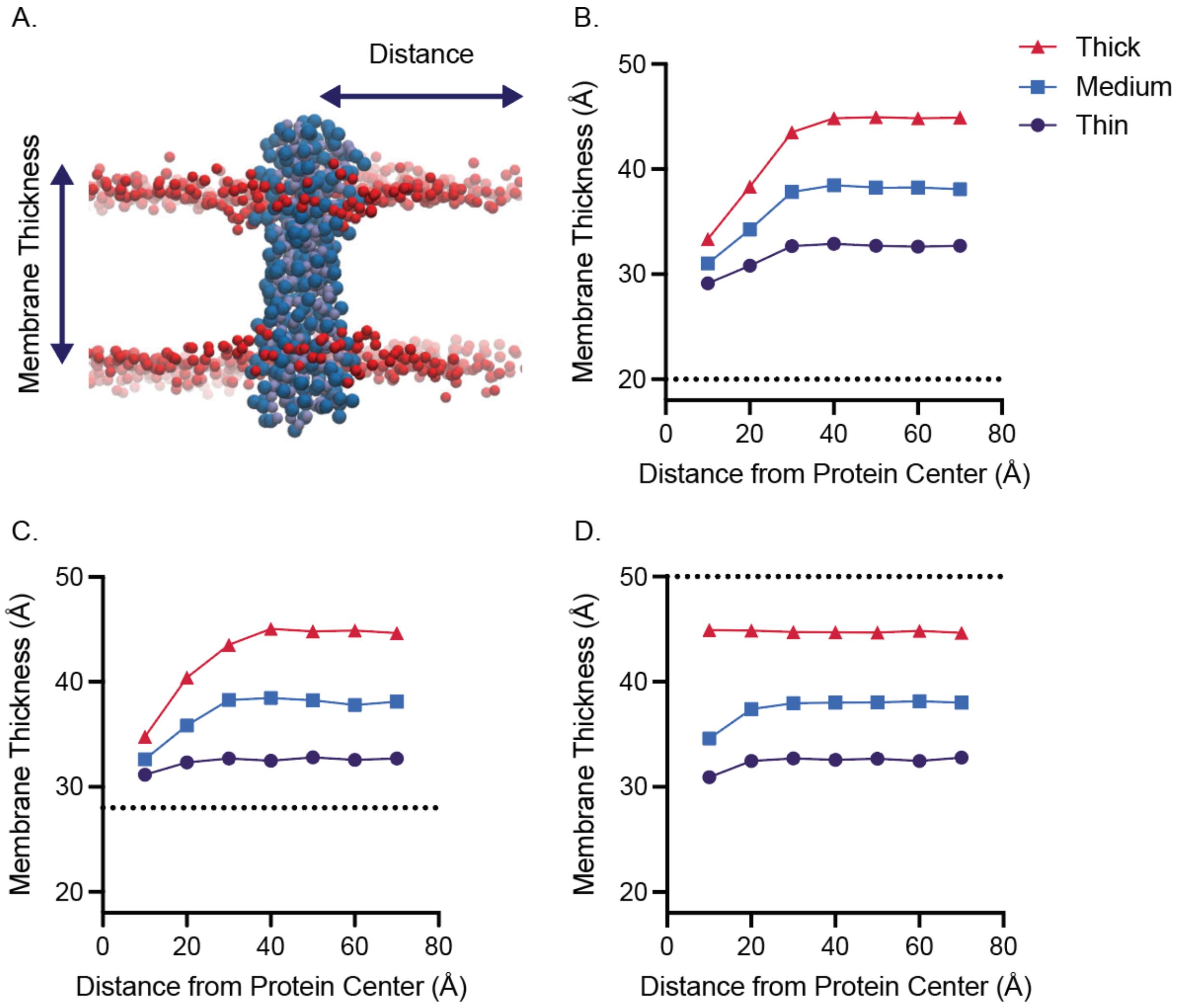

Supplementary Figure 1. Membranes must deform to accommodate hydrophobic mismatch. Using MD simulations, the thickness of the membrane was recorded as a function of distance from the protein. (**A**) Snapshot of the simulation of the 20 Å thick protein in DGPC membrane shows local membrane deformation. This corresponds to the thick membrane (red line) in (**B**). The lipid headgroups are shown in red. This analysis was performed for the 20 (**B**), 28 (**C**), and 50 Å (**D**) proteins in thin (DYPC), medium (DOPC), and thick (DGPC) membranes. Horizontal dotted lines represent the hydrophobic thickness of the protein. Data presented in Fig. 1C was generated by subtracting the membrane thickness at 70 Å from membrane thickness at 10 Å from the protein center.

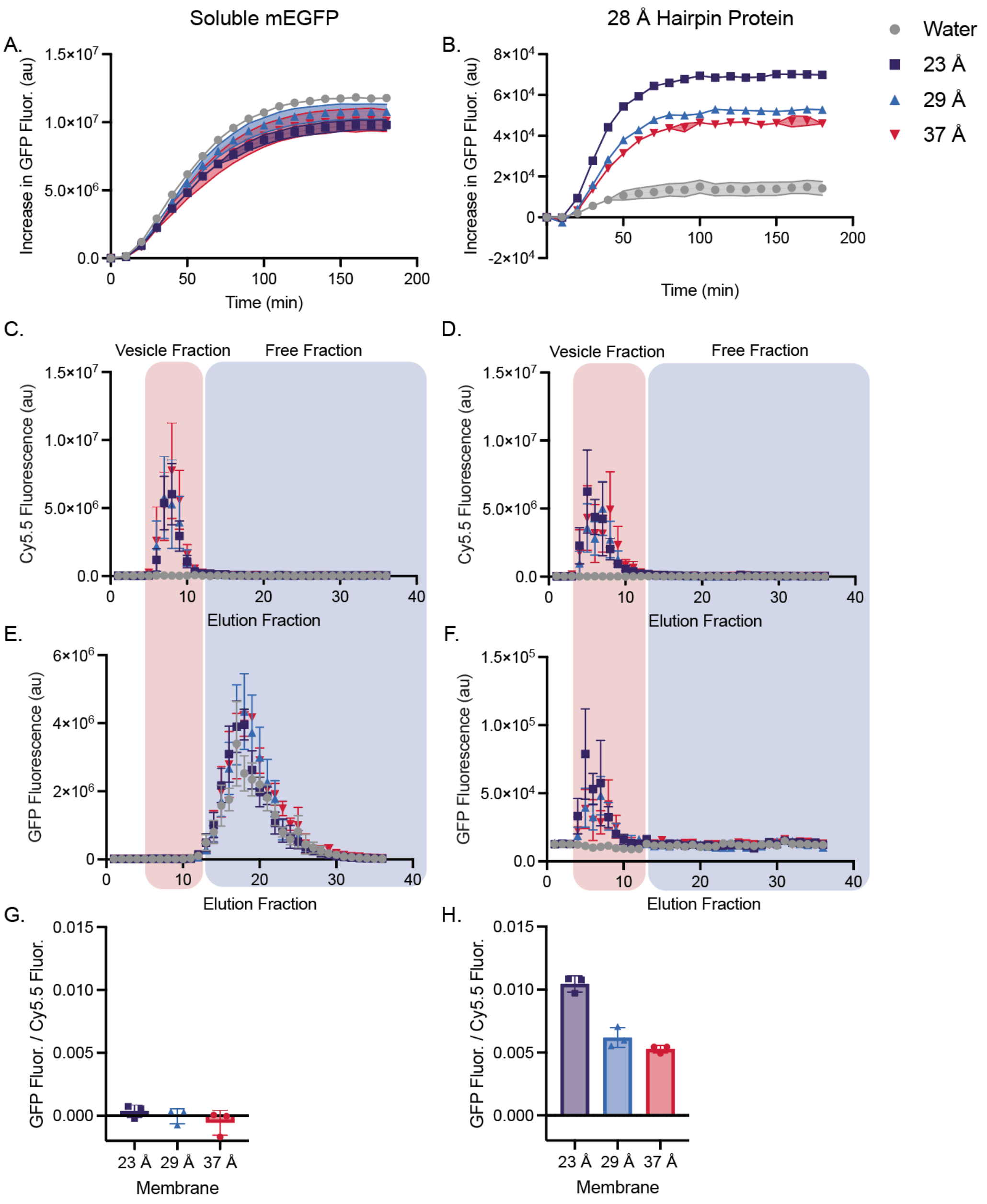

Supplementary Figure 2. Cell-free expressed de novo designed proteins express and insert into synthetic membranes. Soluble mEGFP (left) and the 28 Å hairpin fused to mEGFP (right) were expressed in the presence of water or 23 (14:1 PC), 29 (DOPC), 37 Å (22:1 PC) membranes (10 mM lipid). (**A**, **B**) Background subtracted GFP fluorescence overtime of mEGFP and the 28 Å hairpin. Size exclusion chromatography was then performed on samples to separate vesicle associated and free protein. Vesicles were labeled with Cy5.5 dye enabling vesicle elution fractions to be identified (**C**, **D**). GFP fluorescence was read to observe where protein eluted relative to vesicles (**E**, **F**). Soluble mEGFP eluted in the void volume as it does not associate with the membrane, while GFP fused to the 28 Å hairpin eluted with vesicles. (**G**, **H**) Quantifying the GFP fluorescence relative to the Cy5.5 lipid fluorescence allows for the quantification of GFP associated with lipid. From this analysis, GFP does not associate with membranes and the 28 Å hairpin inserts best into the 23 Å membrane, which mirrors the GFP fluorescence assay in (B). *n=3*, error bars represent S. E. M.

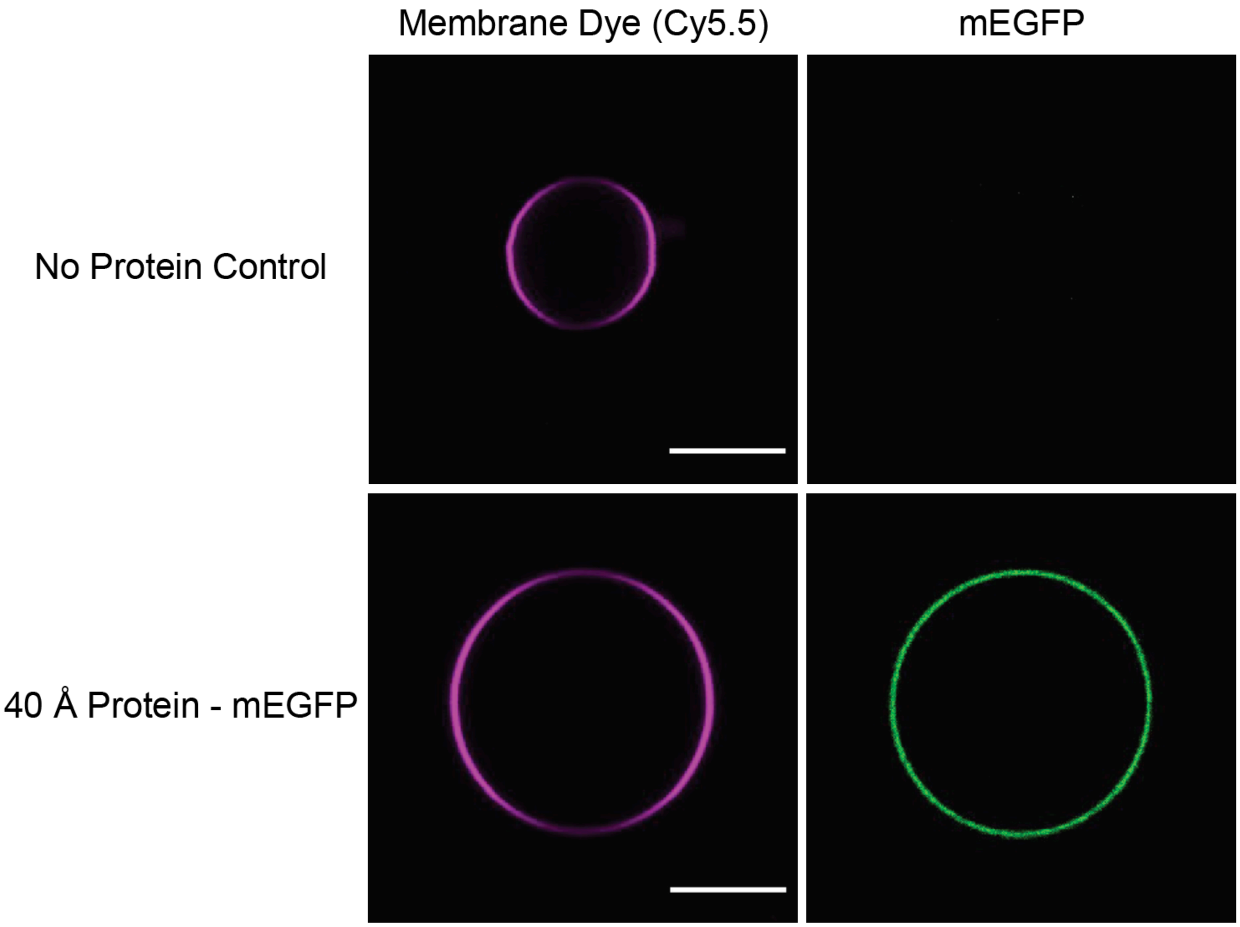

Supplementary Figure 3. Membrane proteins insert in fold into synthetic membranes as assessed by confocal microscopy. Pictured is the 40 Å protein in a DOPC membrane. GUVs were prepared with 99.9 mol% DOPC, 0.1 mol% Cy5.5 conjugated to 18:1 PE. When the 40 Å protein is expressed into synthetic lipids, GFP localizes to the membrane, indicating membrane protein insertion and folding (GFP right, Cy5.5 membrane left). This corresponds to the 29 Å thick membrane and 40 Å thick protein if Fig. 1E. Scale bar 10 µm.

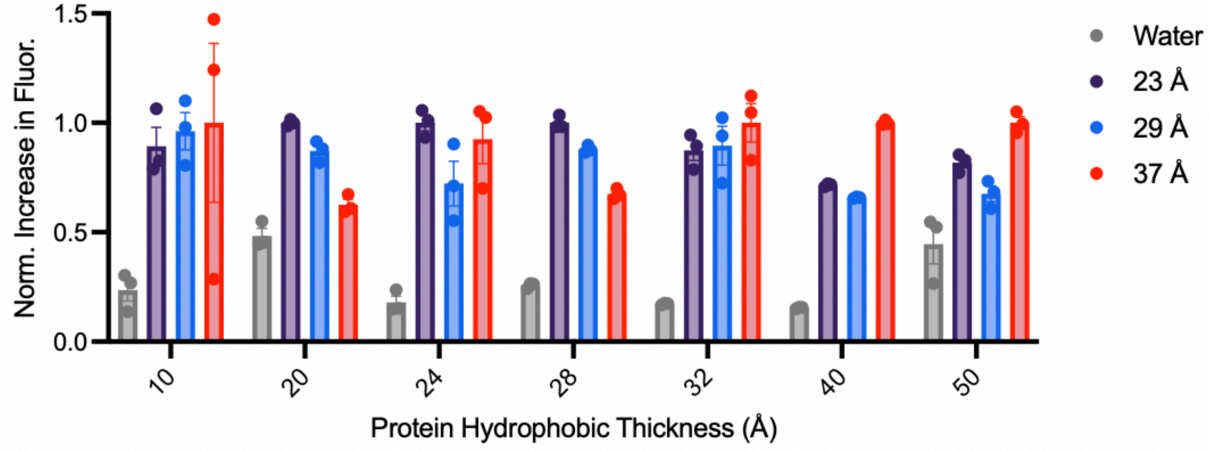

Supplementary Figure 4. Highest increases in GFP fluorescence are observed when hydrophobic mismatch is minimized. Data presented in Figure 1E replotted as a bar graph to display error. Proteins were expressed in the presence of water (no membrane) and 23, 29, and 37 Å thick membranes (14:1 PC, 18:1 PC, and 22:1 PC, respectively). Increase in GFP fluorescence was normalized to the maximum increase for each construct. *n=3*, error bars represent S. E. M.

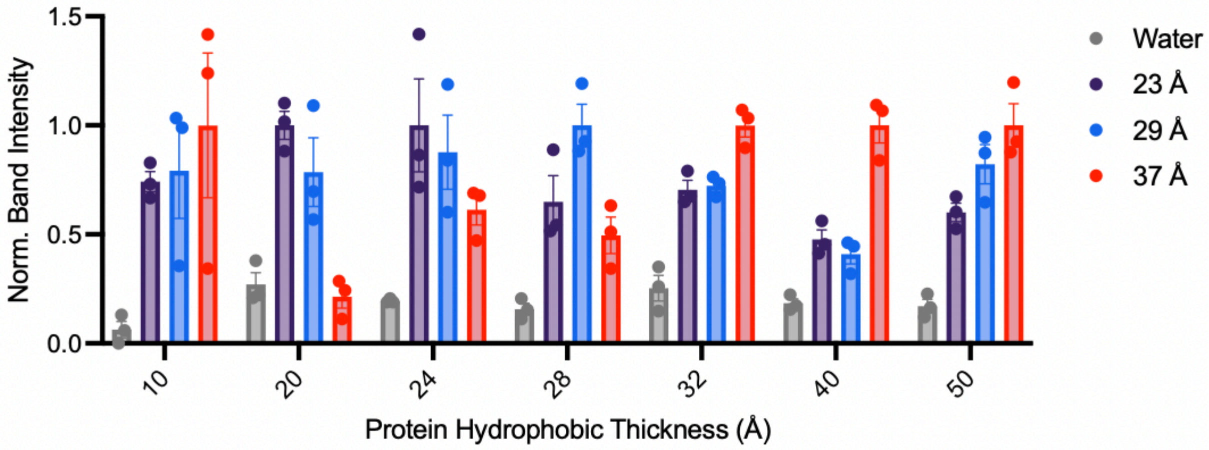

Supplementary Figure 5. Highest protein expression levels are observed when hydrophobic mismatch is minimized. Western blots were performed on samples represented in Fig. 1E. Proteins were expressed in the presence of water (no membrane) and 23, 29, and 37 Å thick membranes (14:1 PC, 18:1 PC, and 22:1 PC, respectively). Protein band intensity was normalized to the most intense band for each construct. *n=3*, error bars represent S. E. M.

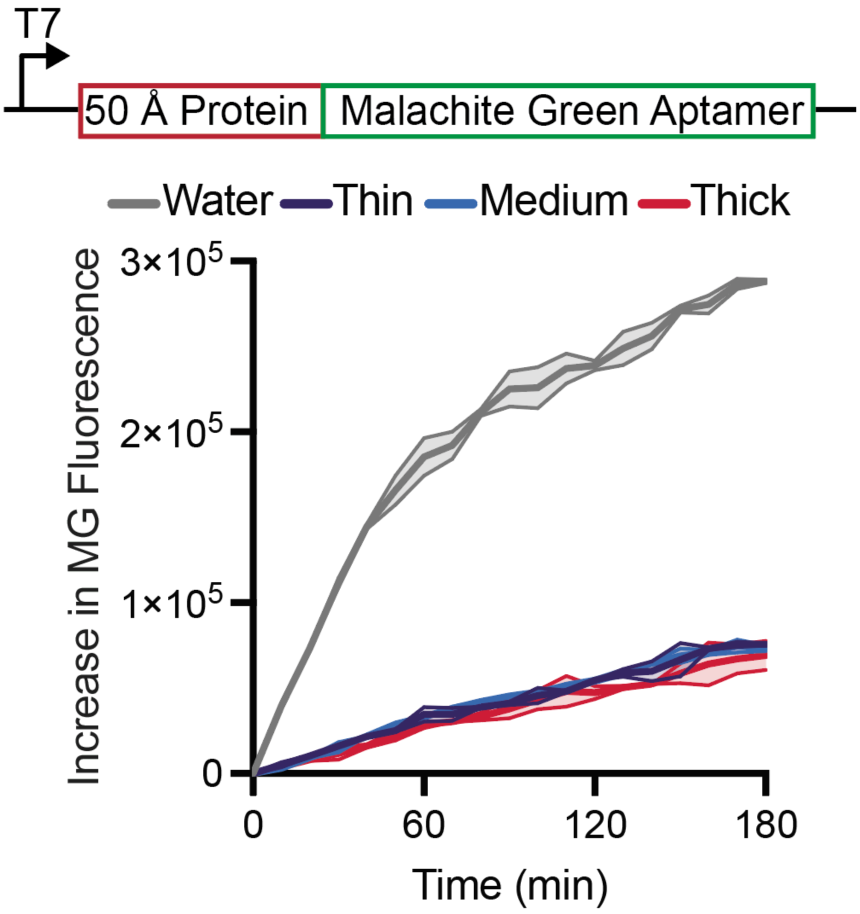

Supplementary Figure 6. Transcription does not change as a function of membrane-protein hydrophobic mismatch. The 50 Å protein was expressed in the presence of water (no membrane), thin (14:1 PC), medium (DOPC), and thick (22:1 PC) membranes and transcription was monitored using the malachite green aptamer. Transcription of the 50 Å protein, as reported by malachite green fluorescence, decreases with the addition of lipid vesicles, but does not change as a function of hydrophobic mismatch. *n=2*, error bars represent S. E. M.

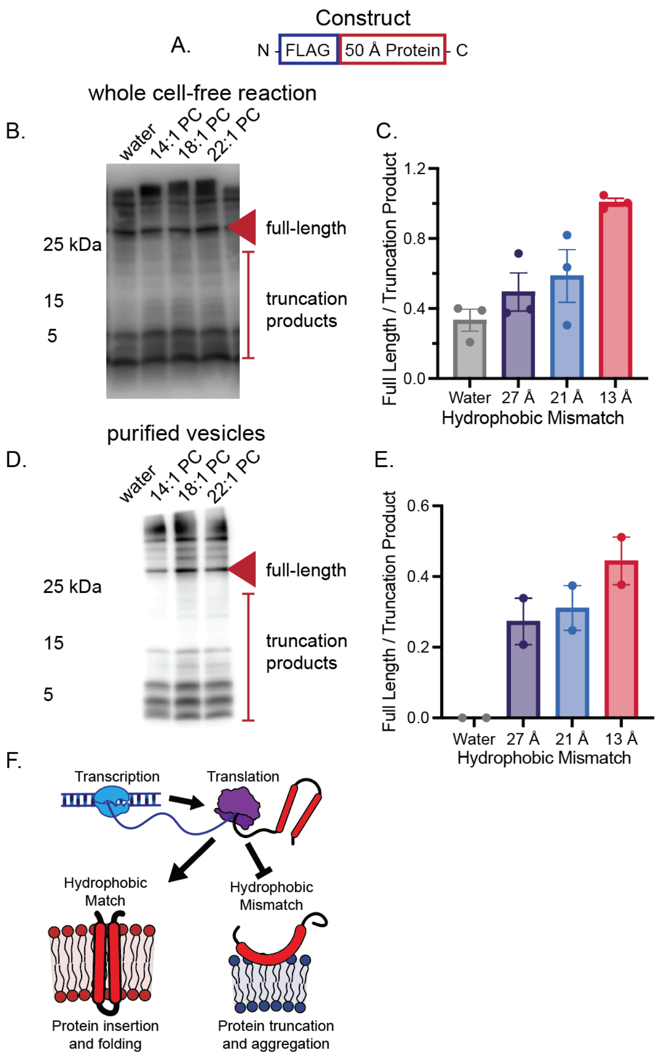

Supplementary Figure 7. Analysis of truncation products via western blot. (**A**) An N-terminal flag tag was added to the 50 Å hairpin protein enabling the detection of all protein products. (**B**) The construct was expressed in the presence of water, 14:1 PC (23 Å), 18:1 PC (29 Å), and 22:1 PC (37 Å) and protein formation was assessed via western blot. (**C**) The proportion of full length 50 Å protein relative to incomplete proteins, increases as hydrophobic mismatch is minimized. Protein expression and truncation products were assessed by performing a Western Blot against a N-terminal FLAG tag. Values represent the mean of 3 independent replicates, error bars represent the S. E. M. (**D, E**) Western blot and quantification of full-length and truncated protein products associated with purified membranes. To observe full-length protein and truncation products associated with vesicles, 1 mol% Biotinyl Cap PE was included in each membrane. 3.5 µL of cell-free reaction mixture was mixed with 0.1 mg of Pierce Streptavidin beads to capture vesicles. The bead mixture was then resuspended and loaded into the gel. The intensity of the full-length product and truncation products, as noted by the labeled arrows, was measured using ImageJ and the ratio of full-length to truncation product intensity. Values represent the mean of 2 independent replicates, error bars represent the S. E. M. (**F**) Schematic illustrating how hydrophobic mismatch may lead to an increase truncated proteins products and subsequent decreased in protein expression.

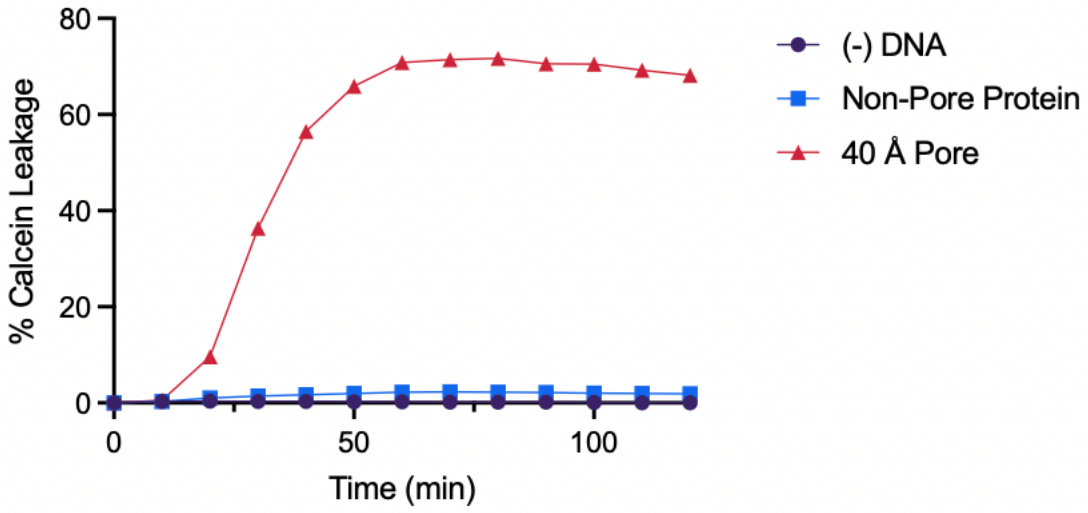

Supplementary Figure 8. The expression and insertion of transmembrane pore proteins enables the release of calcein dye. 50 mM calcein, a self-quenching dye, was encapsulated into DOPC vesicles. Vesicles were then added to a cell free reaction without DNA, or DNA encoding the 40 Å hairpin (non-pore protein) or pore protein. Upon expression and insertion of the 40 Å pore protein, an increase in fluorescence because of calcein leakage and subsequent dequenching was observed. This demonstrates that calcein leakage is specific to pore protein expression and integration into vesicle membranes, and not due to interactions with PURExpress or insertion of non-pore proteins.

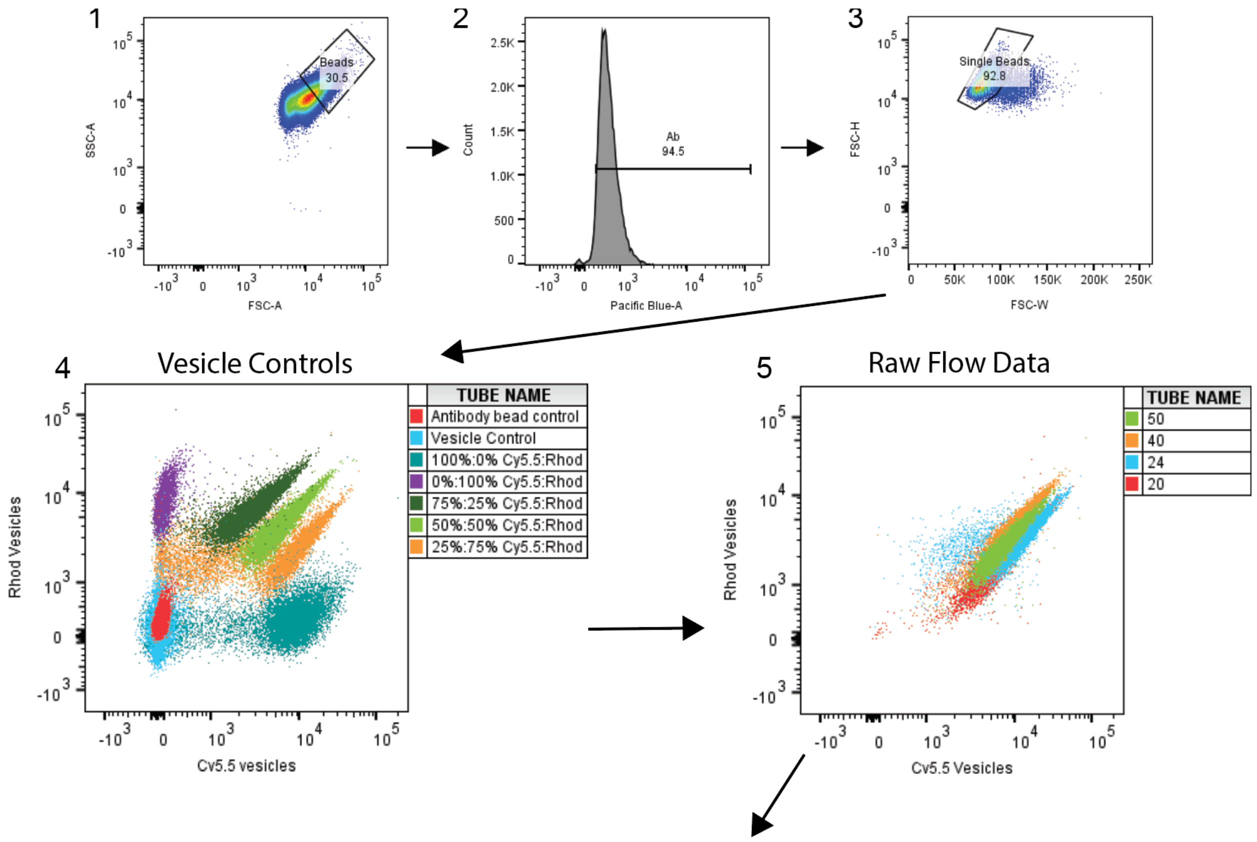

6 $$Enrichment\ in\ thick\ membrane = {MFI\ (Rhodamine, 22{:}1\ PC)}/{MFI\ (Cy5.5, 14{:}1\ PC)}$$

Supplementary Figure 9. Gating strategy for bead-based protein sorting assays (Figure 3C and 3D). Magnetic protein A/G beads were bound with Pacific Blue conjugated anti-FLAG antibody. Beads were first gated on sizes (1) to analyze larger beads and reduce analysis of unbound vesicles, then gated by the presence of antibody (2). Beads were then gated to analyze single beads (3) and then analyzed for Rhodamine and Cy5.5 fluorescence. First, vesicle controls were analyzed to confirm that the assay was able to detect differences in protein insertion into different vesicle populations. To do this, the 40 Å protein was expressed into DOPC vesicles labeled with either 0.1 mol% 18:1 PE Rhodamine or 0.1 mol% 18:1 PE Cy5.5. By expressing the protein into both samples composed of the same lipid, only differing in dye, we could assume that the protein insertion into both was equivalent. Each vesicle set was then mixed in defined ratios (100% Cy5.5, 0% Rhodamine; 0% Cy5.5, 100% Rhodamine; 75% Cy5.5, 25% Rhodamine; 50% Cy5.5, 50% Rhodamine; 25% Cy5.5, 75% Rhodamine), bound to beads, and then analyzed via flow cytometry. A shift in Rhodamine and Cy5.5 fluorescence which corresponded to the initial ratio was observed. This experiment was then repeated with rhodamine labeled 22:1 PC vesicles and Cy5.5 labeled 14:1 PC vesicles. 22:1 PC and 14:1 PC vesicles were mixed 1:1 and each protein was expressed with this mixture of vesicles. Samples were then bound to beads and analyzed via flow cytometry (5) and enrichment in the thicker, 22:1 PC membranes was calculated (6).

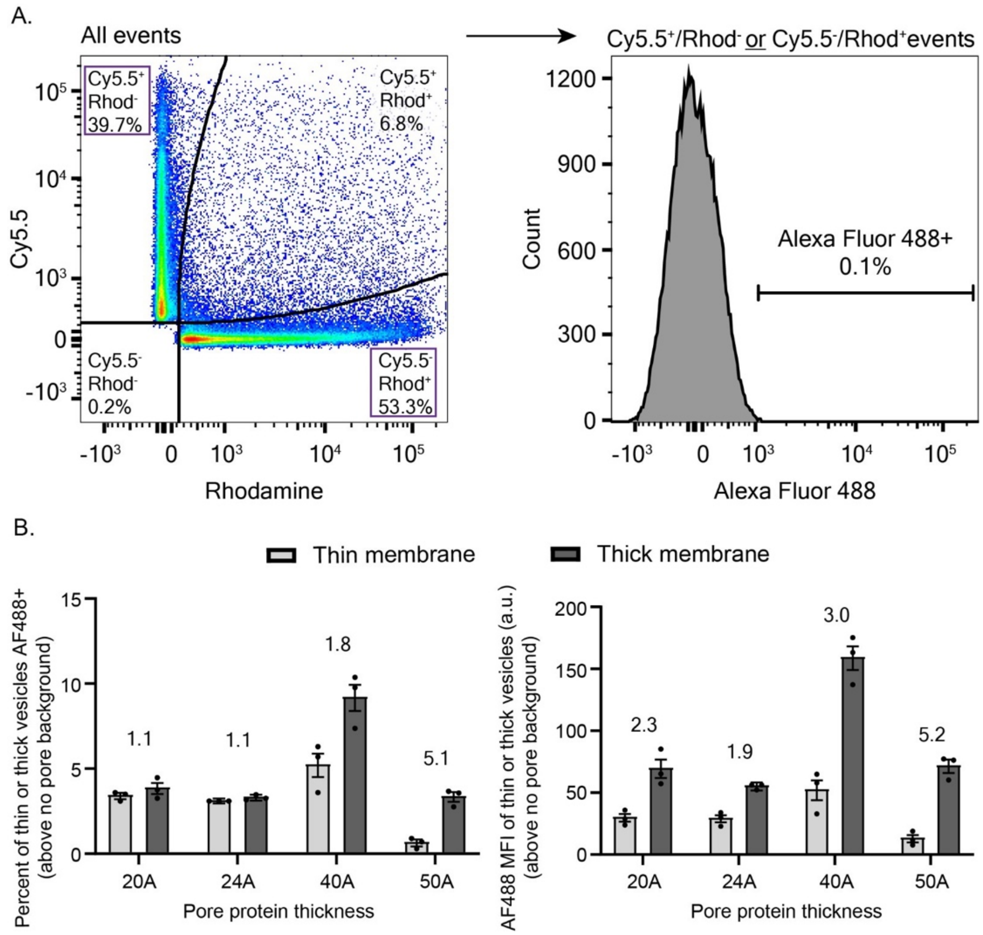

Supplementary Figure 10. Gating strategy and population metrics for pore permeability flow data presented in Figure 3E and 3F. Thin (14:1 PC) and thick (22:1 PC) membrane vesicles were labeled with a single membrane dye, either Cy5.5 or Rhodamine, respectively. (**A**) To analyze subsequent flow cytometry data, events were first gated via a curly quad gating strategy. This identified vesicle populations with only a single membrane dye (left image, top left gate and bottom right gate). A negative control sample without Alexa Fluor 488 dye was used to set the threshold for an Alexa Fluor 488 (AF488) positive signal (right). This gate was then applied to single positive vesicle populations to determine AF488 signal as a measure of functional protein pore insertion. (**B**) Population level metrics of the data presented in Figure 3F. The left figure depicts the percentage of vesicles with a single membrane dye that are also AF488 positive per the gating strategy in (**A**) for different protein pore thicknesses. The right figure depicts the AF488 mean fluorescence intensity (MFI) of vesicles with a single membrane dye for a given pore protein thickness (i.e., the MFI is calculated from samples gated per only the left image in (**A**). In both cases, the data are background subtracted; the background was determined from vesicles incubated with biocytin but not a co-expressed pore protein. Data points represent the three replicates for each condition, the bar graphs represent the mean, and the error bars represent the standard deviation. The numbers are ratios of thick membrane average to thin membrane average for a given metric and pore protein thickness. We take these ratios to

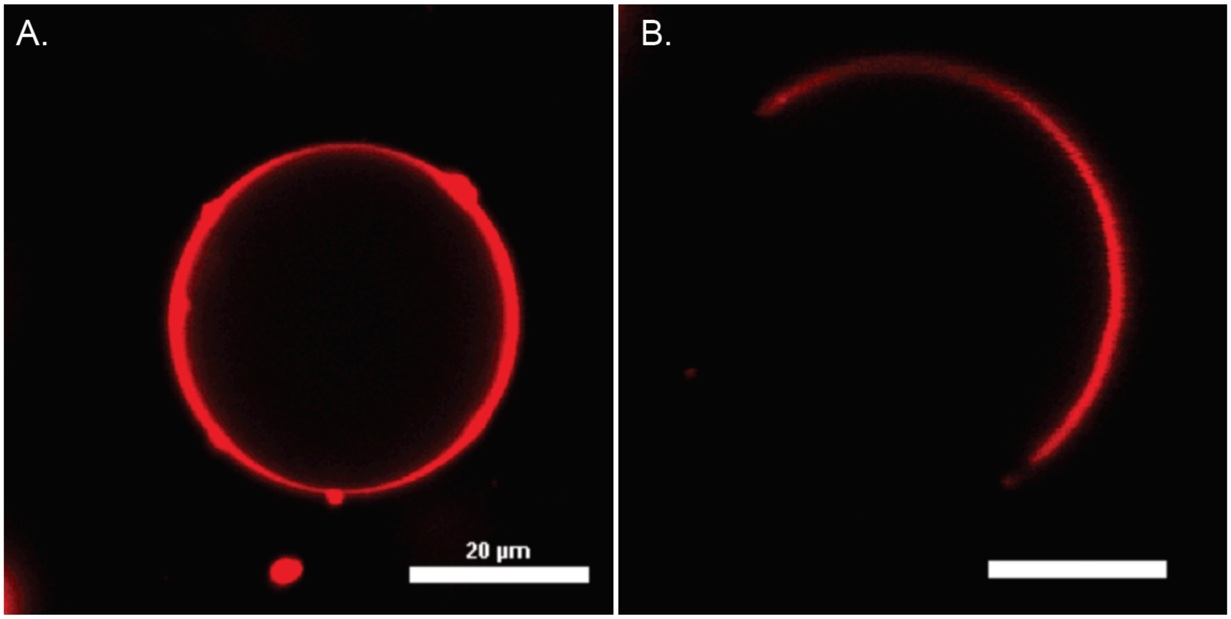

Supplementary Figure 11. Fluorescent microscopy of giant unilamellar vesicles demonstrates that lipid mixtures do not form microdomains. (**A**) Vesicles composed of 42.5 mol% 14:1 PC/27.5 mol% DPPC/30 mol% Chol (composition used in this study) and (**B**) 40 mol% 14:1 PC/40 mol% DPPC/20 mol% Chol. Both membranes in (**A**) and (**B**) were labeled with 0.1 mol% 18:1 PC Rhodamine, which localized to the lipid disordered phase. Exclusion of dye in a region of the membrane, as seen in (**B**), indicates the presence of microdomain formation, a property often seen in previous studies. By increasing the cholesterol content and decreasing the amount of DPPC, membranes that do not exhibit microdomain formation (**A**) are formed. Samples with higher cholesterol content used to make vesicles in panel A do not exhibit microdomain formation. Scale bars are 10 µm.

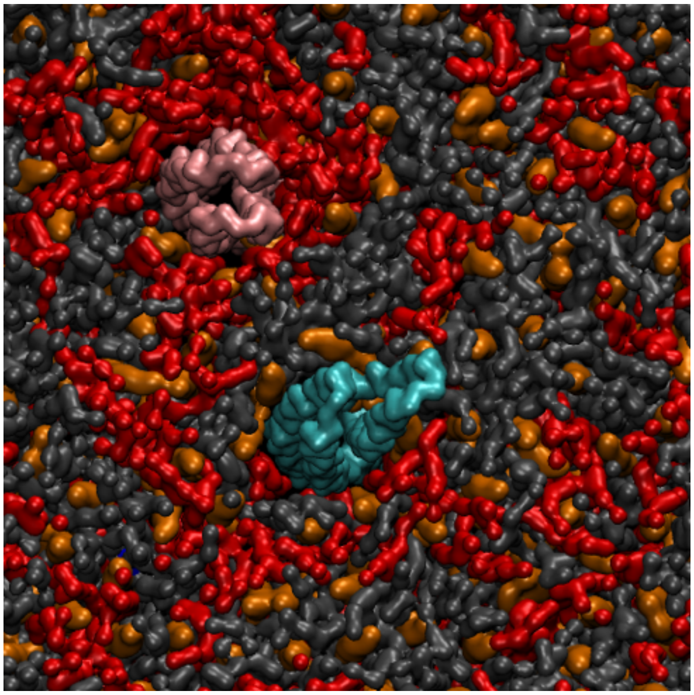

Supplementary Figure 12. Simulation of 20 and 50 Å proteins in phase separating membranes. DYPC lipid (red) nucleates around the 20 Å hairpin protein (pink). The 50 Å protein (blue) is in contact with DPPC (grey) and cholesterol (gold) more often than DYPC (red). Further, proteins are apart from one another. Representative image is from Movie S1. The membrane is composed of 42.5 mol% DYPC/27.5 mol% DPPC/30 mol% Cholesterol.

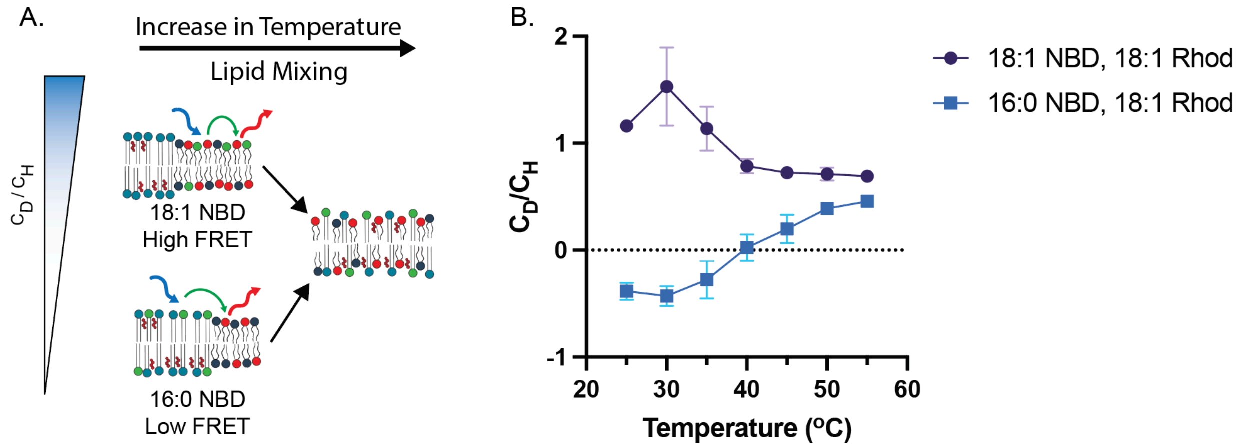

Supplementary Figure 13. Lipid organization can be detected by lipid-lipid FRET. (**A**) Differences in C_D/C_H can be measured when incorporating either 18:1 or 16:0 PE with headgroup conjugated NBD (ex. 460 nm /em. 535 nm) in vesicles composed of 42.5 mol% 14:1 PC/27.5 mol% DPPC/30 mol% Cholesterol and 18:1 PE Rhodamine (ex. 560 nm/em. 590 nm). Lipid-lipid FRET was used to validate C_D/C_H as lipid phase separation has been well characterized and is more well defined. (**B**) 18:1 PE NBD resides in the liquid disordered lipid phase, with 18:1 Rhodamine, and this has a higher C_D/C_H compared to vesicles with 16:0 NBD, which resides in the liquid ordered phase, farther from 18:1 Rhodamine (lower C_D/C_H). Upon increasing temperature, C_D/C_H converge, indicated that lipids intermix at higher temperatures due to increases in membrane fluidity and subsequent lipid miscibility.

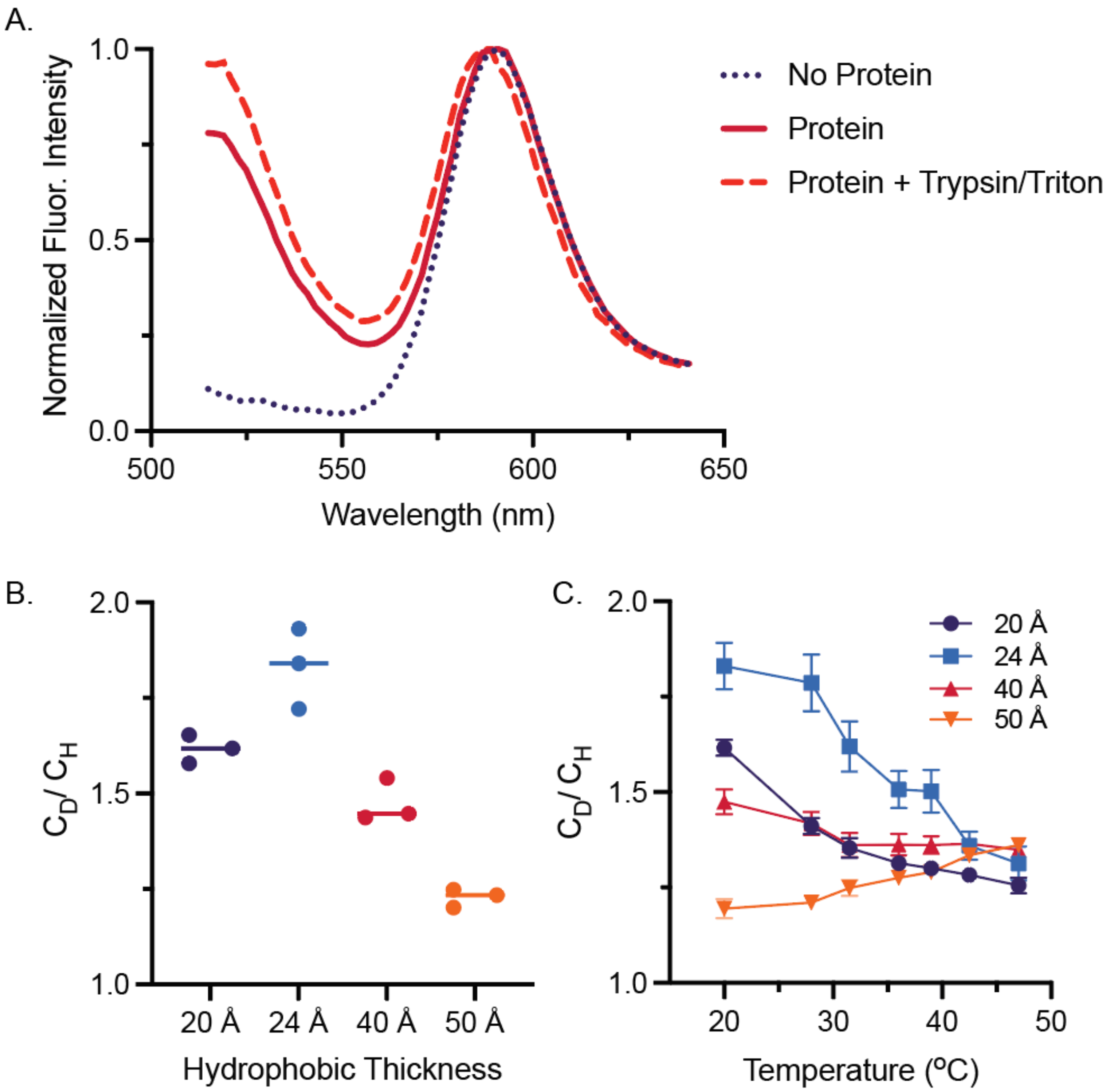

Supplementary Figure 14. SNAP conjugated fluorophores enable lipid-protein FRET and protein-lipid interactions to be probed in vitro. (**A**) Spectra of SNAP conjugated protein in a homogenous DOPC membrane containing 0.1 mol% 18:1 PE conjugated to Rhodamine before and after the addition of trypsin and triton. The dequenching of AF488 after the addition of triton and trypsin indicates that lipid-protein FRET is occurring. (**B**) At room temperature, C_D/C_H values are higher for membrane proteins with shorter transmembrane domains, indicating that the shorter the transmembrane domain, the closer the protein is to rhodamine on average. (**C**) Upon increasing temperature, C_D/C_H for all constructs converges to the same value, indicating that lipid and proteins mixing can occur at elevated temperatures.

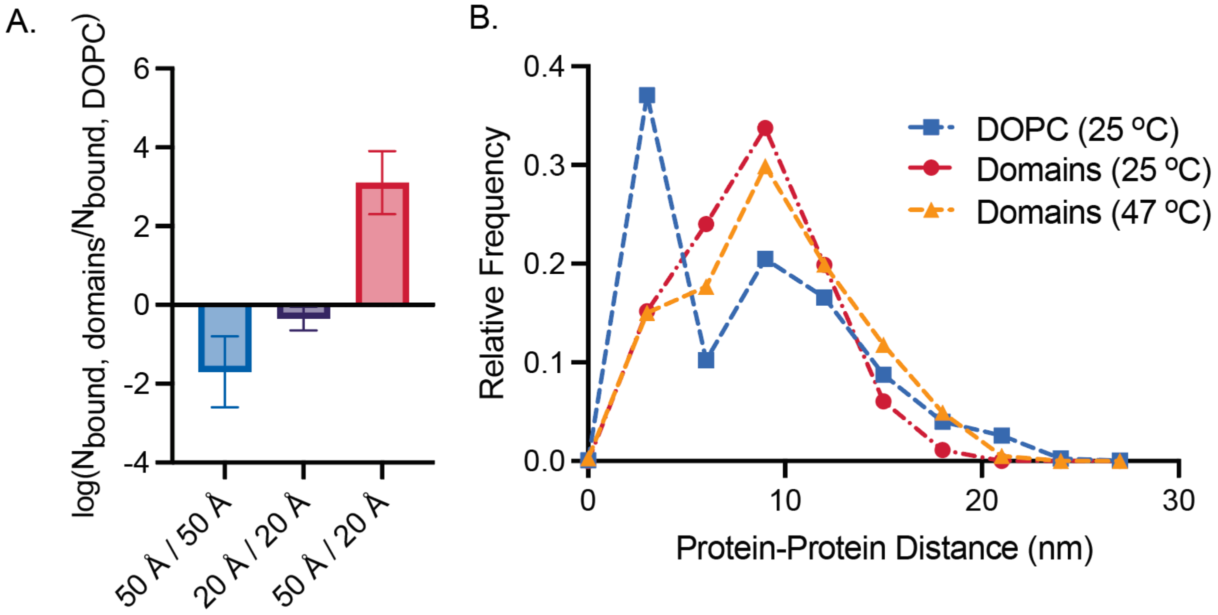

Supplementary Figure 15. MD simulations predict protein-protein in synthetic membranes. (**A**) Simulations of homotypic and heterotypic pairs of the 50 Å and 20 Å proteins were run in single component (DOPC, no domains) or tertiary membranes (42.5 mol% DyPC/27.5 mol% DPPC/30 mol% Cholesterol, domains). The changes in protein association were quantified by calculating the pseudo free energy difference between the protein-protein tertiary and single component membranes as -log($N_{bound,ternary}$/$N_{bound,single}$), where N is the number of states which we considered bound. Proteins were considered bound if their center-to-center distance was below 3 nm. Standard errors were calculated from simulation replicates. Negatives values represent enhanced protein-protein contact formation in ternary membranes compared to single component membranes. These data demonstrate that proteins of different transmembrane domain lengths are farther apart in the ternary lipid mixture relative to the homogenous membrane but proteins of equal transmembrane domain length are closer together in ternary membranes relative to homogenous membranes.[1] (**B**) Protein-protein distance of 20 and 50 Å proteins in DOPC and domain forming (42.5 mol% DyPC/27.5 mol% DPPC/30 mol% Cholesterol) lipid mixtures at 25°C and 47°C. At 25 °C, protein-protein distance between the 20 and 50 Å hairpin is on average smaller in homogenous, single component DOPC membranes compared to membranes composed of 42.5 mol% DyPC/27.5 mol% DPPC/30 mol% Cholesterol. At 47°C, protein-protein distance decreases in membranes composed of 42.5 mol% DyPC/27.5 mol% DPPC/30 mol% Cholesterol due to increased lipid mixing. Histograms were generated from 3 independent simulations. Bin size is 3 nm, the approximate protein center to center distance when bound.

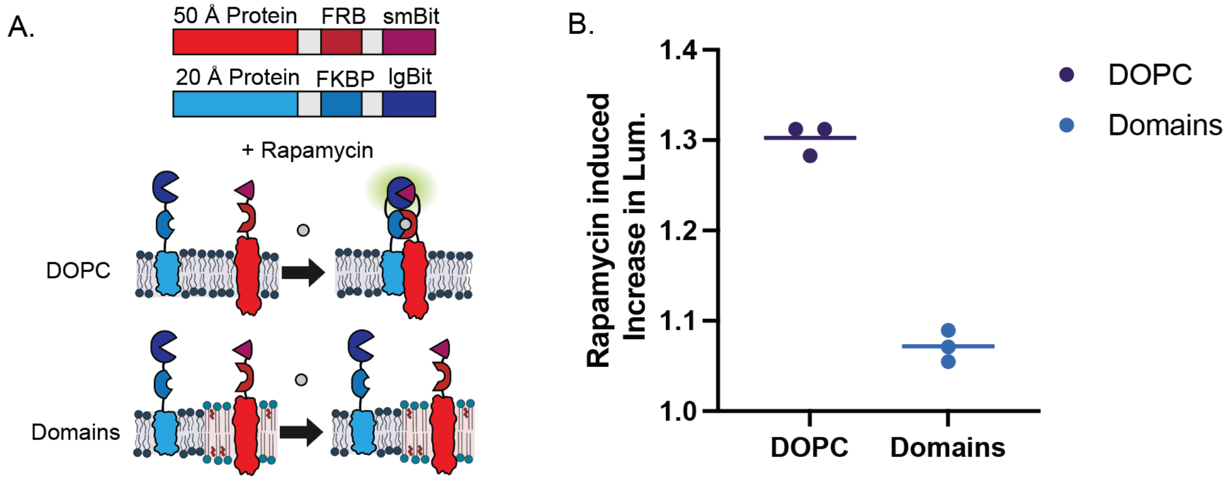

Supplementary Figure 16. Proteins are more prone to dimerization in DOPC membranes compared to membranes prone to phase separation. (**A**) FRB and FKBP were fused to the C-terminus of the 20 and 50 Å protein, respectively. Addition of rapamycin forced proteins to dimerize and NanoBit to become reconstituted, enabling the evaluation of protein-protein interactions. (**B**) An increase in luminescence, because of the protein dimerization, was observed in DOPC membranes, but not membranes prone to domain formation (42.5 mol% 14:1 PC/27.5 mol% DPPC/30 mol% Cholesterol).

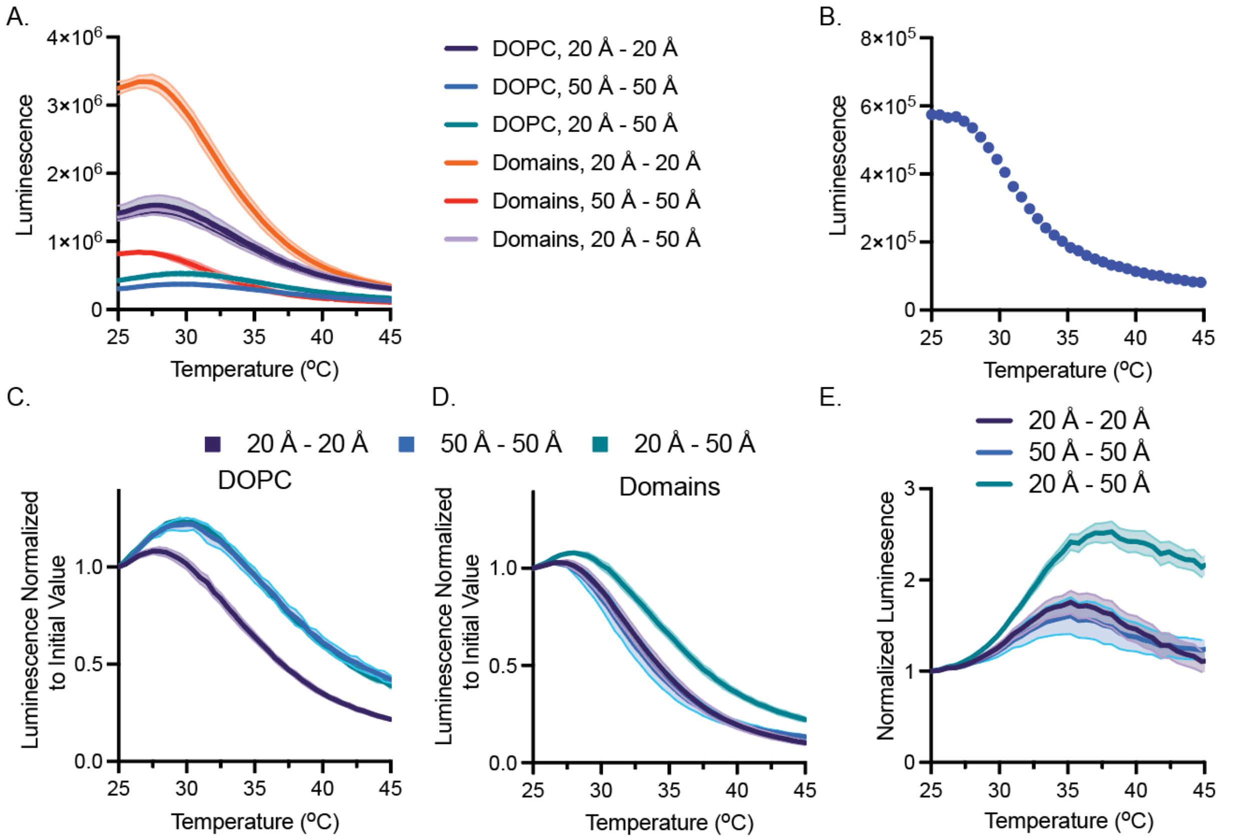

Supplementary Figure 17. Analysis of split luciferase reconstitution in response to domain dissolution via heating. (**A**) Raw luminescence data of split luciferase constructs in DOPC and ternary lipid membranes (42.5% 14:1 PC/27.5% DPPC/30% Chol). Luminescence values differ due to differences in expression. Values decrease with increasing temperature, as luciferase is less efficient at elevated temperatures as demonstrated by a luminescence vs temperature for soluble NanoBit (**B**). By normalizing by the initial value, we can compare how luminescence for each combination of proteins changes in (**C**) DOPC and (**D**) ternary lipid membranes (42.5% 14:1 PC/27.5% DPPC/30% Chol). For domain forming mixtures, transmembrane homodimers decrease more quickly than the heterodimer case. This suggests that homodimers interact less a result of lipid demixing and heterodimers are able to interact more and thus reconstitute luciferase. Data used to generate Fig. 3H. *n=3*, error bars represent the S. E. M. (**E**) An increase in normalized luminescence is also observed in for ternary lipid membranes (42.5% 14:1 PC/27.5% DPPC/30% Chol) containing the 20 and 50 Å protein relative to membranes containing either 20 Å-20 Å or 50 Å-50 Å proteins. This plot was generated by dividing the data presented in (D) with the luminescence data in (B) to correct for changes in luciferase function with temperature and normalizing to the luminescence value at room temperature. *n=3*, error bars represent the S. E. M.

Supplementary Table 1. Physical features of lipids used in this study. Reported transition temperatures (T_m) are listed by Avanti Polar Lipids, from which the lipids were purchased.

**Lipid**	**T_m (°C)**	**Thickness (Å)**
14:1 PC	n/a	23.4[2]
18:1 PC (DOPC)	-17	29.3[2]
22:1 PC	13	37.2[2]
16:0 PC (DPPC)	41	34.4[3]

Supplementary Table 2. Sequences of protein designs. Transmembrane domains are marked in boldface.

Design	Hydrophobic Length (Å)	Sequence
PL421	10	MTRKEIIEKLEKSLRRQ**KELAERLLILLLLLLR**LLHELLELLRRLEELQRRGSSDEEVHELLRRIIELVERII**YLVIFIIALVREIIK**LAEHQRRLVEELKKQD
PL422	20	MTRKEIIEKLEKSLRRQK**KLARFLLILLLLLLALLLE**LLELLRRLEELQRRGSSDEEVHELLRRIIELVE**YIILLVLFIIVLVRIIIK**LAEHQRRLVEELKKQD
PL191	24	MTRTEIIRELERSL**REQRVLAIFLLALLIVLLW**LLQQLKELLRELERLQREGSSDEDVRELLREIKELVENIV**YLVIIIMVLVLVIIALARTQK**YLVEELKRQD
PL145	28	MTRTEIIRELERSL**RLQLVLAIFLLGLLIVLLW**LLQQLKELLRELERLQREGSSDEDVRELLREIKELVENIV**YLVIIIMVLVLVIIALTVTQK**YLVEELKRQD
PL424	32	MTRTEIIT**RLSFSLLLQLVLAIFLLALLIVLLW**LLQQLKELLRELERLQREGSSDEDVRELLREIKELVENIV**YLVIIIMVLVLVIIALAVLQMYLVR**ELKRQD
PL193	40	MTRTEIIT**RLSFSLLLQLVLAIFLLALLIVLLVLLIY**LKELLRELERLQREGSSDEDVRELLREIK**WLVIVIVALVIIIMVLVLVIIALAVLQMYLVR**ELKRQD
PL209	50	MVLSHHFG**KEFASATMTRTEIITRLSFSLLLQLVLAIFLLALLIVLLVLLIVLMILLIALEY**LQKEGSSDEDV**KELLVLIMILVIVIVALVIIIMVLVLVIIALAVLQMYLVR**ELKRQD
TMH4C4-20	20	MSAEELLRRSRE**YLKKVAKFQLVIALVFLILLEILSR**RSEELIRELEEKGAASEAELARMKQQHMTA**YLQAALTAWEIISKSLIALLLL**QQNQLNLELNTDTDKNVAEELLRRSREYLK**KVAKKQLVIAFVFLILLEILSR**RSEELIRELEEKGAASEAELARMKQQHMTA**YLQAALTAWEIISKSLIALLLL**QQNQLNLELRH
TMH4C4-24	24	MSAEELLRRSRE**YLKKVAKIQLVIALVFLILLIILSR**RSEELIRELEEKGAASEAELARMKQQHMTA**YLQAALTAWEIISKSVIALLLL**QQNQLNLELNTDTDKNVAEELLRRSREYLK**KVAKIQLVIAFVFLILLIILSRRSEELIR**ELEEKGAASEAELARMKQQHMTA**YLQAALTAWEIISKSRIALLLL**QQNQLNLELRH
TMH4C4-40	40	MSAEELLRRSRK**YLILVALIQLVIAFVFLILLILLSWLSWLLIR**ELEEKGAASEAELARM**KIQMMTAYLQAALTAWEIIVKAVIALLLLR**QNQLNLELNTDTDKNVAEELLRRSR**KYLIIVALIQLVIAFVFLILLILLSWKSWELIR**ELEEKGAASEAELARMKMQVMLA**YLQAALTAWEIIAKSVIALLLLL**QNQLNLELRH
TMH4C4-50	50	MSAEELLRWSRI**YLVIVALIQLVIAFVFLILLILLSWLSLVLIW**ELEEKGAASEAELA**RMILQVMTAYLQAALTAWEII**

		**AKVVIALLLLVVNQLNL**ELNTDTDKNVAEELLR**RSLLYLIMVALIQLVIAFVFLILLILLSWISLLLIW**ELEEKGAASEAELA**RMAIQLMIAYLQAALTAWEIIAKSVIALLLLIL**NQLNLELRH

Supplementary Table 3. Table of lipid compositions used.

Figure	Panel	Composition
Fig. 1	b	DYPC (di-C12:1-C14:1 PC), DOPC (18:1 PC), DGPC (di-C20:1-C22:1) (simulation); 216 MARTINI lipids per leaflet
	c	DYPC, DOPC, DGPC (simulation); 216 MARTINI lipids per leaflet
	e	Water, 14:1 PC, DOPC, 22:1 PC
	f	Comparison of simulated membrane compression in DYPC, DOPC, DGPC to GFP fluorescence in 14:1 PC, DOPC, 22:1 PC membranes
	g	Water, 14:1 PC, DOPC, 22:1 PC
Fig. 2	c	14:1 PC
	d	22:1 PC
	f	14:1 PC and 22:1 PC
	h	14:1 PC and 22:1 PC
Fig. 3	a	42 mol% DYPC/28 mol% DPPC (16:0 PC)/30 mol% Cholesterol (simulation) or 138 DYPC, 92 DPPC and 99 cholesterol molecules per leaflet
	c	42.5 mol% 14:1 PC/27.5 mol% DPPC/30 mol% Cholesterol
	d	42.5 mol% 14:1 PC/27.5 mol% DPPC/30 mol% Cholesterol
	f	42 mol% DYPC/28 mol% DPPC/30 mol% Cholesterol and DOPC (simulation) or 138 DYPC, 92 DPPC and 99 cholesterol molecules per leaflet and 326 DOPC molecules per leaflet
	h	42.5 mol% 14:1 PC/27.5 mol% DPPC/30 mol% Cholesterol and DOPC
Supplementary Fig. 1	b-d	DYPC, DOPC, DGPC (simulation); 216 MARTINI lipids per leaflet
Supplementary Fig. 2	a-h	Water, 14:1 PC, DOPC, 22:1 PC
Supplementary Fig. 3		99.9 mol% DOPC, 0.1 mol% 18:1 PE Cy5.5
Supplementary Fig. 4		Water, 14:1 PC, DOPC, 22:1 PC
Supplementary Fig. 5		Water, 14:1 PC, DOPC, 22:1 PC
Supplementary Fig. 6		Water, 14:1 PC, DOPC, 22:1 PC
Supplementary Fig. 7		Water, 14:1 PC, DOPC, 22:1 PC
Supplementary Fig. 8		DOPC
Supplementary Figure 9		99.9 mol% 14:1 PC, 0.1 mol% 18: 1 PE Cy5.5; 99.9 mol% 22:1 PC, 0.1 mol% 18:1 PE Rhodamine
Supplementary Figure 10	a, b	99.9 mol% 14:1 PC, 0.1 mol% 18: 1 PE Cy5.5; 99.9 mol% 22:1 PC, 0.1 mol% 18:1 PE Rhodamine

Supplementary Figure 11	a	42.5 mol% 14:1 PC/27.5 mol% DPPC/30 mol% Chol + 0.1 mol% 18:1 PE Rhodamine
	b	40 mol% 14:1 PC/40 mol% DPPC/20 mol% Chol + 0.1 mol% 18:1 PE Rhodamine
Supplementary Figure 12		42 mol% DYPC/28 mol% DPPC/30 mol% Cholesterol (simulation) or 138 DYPC, 92 DPPC and 99 cholesterol molecules per leaflet
Supplementary Figure 13	b	DOPC and 42.5 mol% 14:1 PC/27.5 mol% DPPC/30 mol% Cholesterol with 0.1 mol% 18:1 PE Rhodamine and either 0.1 mol% 18:1 PE or 16:0 PE NBD
Supplementary Figure 14	a	99.9 mol% DOPC, 0.1 mol % 18:1 PE Rhodamine
	b-c	42.5 mol% 14:1 PC/27.5 mol% DPPC/30 mol% Cholesterol with 0.1 mol% 18:1 PE Rhodamine
Supplementary Figure 15		42 mol% DYPC/28 mol% DPPC/30 mol% Cholesterol and DOPC (simulation) or 138 DYPC, 92 DPPC and 99 cholesterol molecules per leaflet and 326 DOPC molecules per leaflet
Supplementary Figure 16	b	DOPC and 42.5 mol% 14:1 PC/27.5 mol% DPPC/30 mol% Cholesterol (Domains)
Supplementary Figure 17	a-e	DOPC and 42.5 mol% 14:1 PC/27.5 mol% DPPC/30 mol% Cholesterol (Domains)

Supplementary Table 4. DNA sequences for all proteins used in this study.

Construct	Sequence (DNA and protein)
10 Å hairpin - mEGFP	ATGGGCTCGACCCGCAAGGAGATCATTGAAAAGTTGGAGAAATCCCTTCG<br>TCGTCAAAAAGAGTTGGCGGAACGCCTTTTGATTCTTCTGTTGTTGTTATT<br>GCGTTTATTACATGAGTTGCTTGAGCTTTTGCGCCGTCTGGAAGAATTGCA<br>GCGTCGCGGGTCGTCAGATGAGGAGGTGCATGAACTTCTGCGTCGCATTA<br>TTGAATTGGTCGAGCGCATCATTTATCTTGTCATCTTTATCATTGCTCTGGT<br>ACGCGAAATTATCAAACTTGCAGAGCACCAGCGTCGTTTGGTAGAAGAGC<br>TTAAAAAGCAGGACGGTAGCAGCGGATCCATGGTGAGCAAGGGCGAGGA<br>GCTGTTCACCGGGGTGGTGCCCATCCTGGTCGAGCTGGACGGCGACGTAA<br>ACGGCCACAAGTTCAGCGTGTCCGGCGAGGGCGAGGGCGATGCCACCTA<br>CGGCAAGCTGACCCTGAAGTTCATCTGCACCACCGGCAAGCTGCCCGTGC<br>CCTGGCCCACCCTCGTGACCACCCTGACCTACGGCGTGCAGTGCTTCAGC<br>CGCTACCCCGACCACATGAAGCAGCACGACTTCTTCAAGTCCGCCATGCC<br>CGAAGGCTACGTCCAGGAGCGCACCATCTTCTTCAAGGACGACGGCAACT<br>ACAAGACCCGCGCCGAGGTGAAGTTCGAGGGCGACACCCTGGTGAACCG<br>CATCGAGCTGAAGGGCATCGACTTCAAGGAGGACGGCAACATCCTGGGG<br>CACAAGCTGGAGTACAACTACAACAGCCACAACGTCTATATCATGGCCGA<br>CAAGCAGAAGAACGGCATCAAGGTGAACTTCAAGATCCGCCACAACATC<br>GAGGACGGCAGCGTGCAGCTCGCCGACCACTACCAGCAGAACACCCCCA<br>TCGGCGACGGCCCCGTGCTGCTGCCCGACAACCACTACCTGAGCACCCAG<br>TCCAAGCTGAGCAAAGACCCCAACGAGAAGCGCGATCACATGGTCCTGC<br>TGGAGTTCGTGACCGCCGCCGGGATCACTCTCGGCATGGACGAGCTGTAC<br>AAGTAA
20 Å hairpin - mEGFP	ATGGGCTCGACTCGCAAGGAAATCATTGAGAAGTTAGAAAAGAGCCTGC<br>GTCGCCAGAAGAAACTGGCACGTTTCCTTTTAATCTTACTTTTACTGTTGT<br>TAGCTCTGCTGCTTGAATTATTGGAGCTTTTGCGCCGTCTGGAAGAGTTGC<br>AGCGTCGCGGCTCAAGTGACGAAGAAGTCCATGAATTATTACGCCGTATC<br>ATTGAGCTTGTGGAATATATTATCCTTCTGGTGTTGTTCATCATCGTACTT<br>GTCCGCATCATCATCAAATTAGCAGAGCATCAACGCCGCTTGGTTGAGGA<br>ACTGAAGAAGCAGGACGGTAGCAGCGGATCCATGGTGAGCAAGGGCGAG<br>GAGCTGTTCACCGGGGTGGTGCCCATCCTGGTCGAGCTGGACGGCGACGT<br>AAACGGCCACAAGTTCAGCGTGTCCGGCGAGGGCGAGGGCGATGCCACC<br>TACGGCAAGCTGACCCTGAAGTTCATCTGCACCACCGGCAAGCTGCCCGT<br>GCCCTGGCCCACCCTCGTGACCACCCTGACCTACGGCGTGCAGTGCTTCA<br>GCCGCTACCCCGACCACATGAAGCAGCACGACTTCTTCAAGTCCGCCATG<br>CCCGAAGGCTACGTCCAGGAGCGCACCATCTTCTTCAAGGACGACGGCAA<br>CTACAAGACCCGCGCCGAGGTGAAGTTCGAGGGCGACACCCTGGTGAAC<br>CGCATCGAGCTGAAGGGCATCGACTTCAAGGAGGACGGCAACATCCTGG<br>GGCACAAGCTGGAGTACAACTACAACAGCCACAACGTCTATATCATGGCC<br>GACAAGCAGAAGAACGGCATCAAGGTGAACTTCAAGATCCGCCACAACA<br>TCGAGGACGGCAGCGTGCAGCTCGCCGACCACTACCAGCAGAACACCCC<br>CATCGGCGACGGCCCCGTGCTGCTGCCCGACAACCACTACCTGAGCACCC<br>AGTCCAAGCTGAGCAAAGACCCCAACGAGAAGCGCGATCACATGGTCCT<br>GCTGGAGTTCGTGACCGCCGCCGGGATCACTCTCGGCATGGACGAGCTGT<br>ACAAGTAA
24 Å hairpin - mEGFP	ATGATGGGCACCAGGACAGAGATCATCAGGGAGCTTGAGCGATCCTTGC<br>GAGAGCAGCGCGTGCTTGCTATTTTCCTCCTGGCGTTGCTCATCGTACTTC<br>TCTGGCTGCTGCAACAACTTAAAGAATTGTTGCGCGAGCTGGAACGGCTG<br>CAAAGAGAAGGTTCATCCGACGAGGATGTAAGAGAATTGCTTAGAGAAA<br>TCAAAGAACTTGTTGAGAACATTGTATATCTGGTAATAATAATCATGGTC<br>CTCGTCCTGGTAATCATAGCCCTGGCAAGAACGCAAAAATACCTTGTCGA<br>AGAGCTGAAGCGGCAGGATGGTAGCAGCGGATCCATGGTGAGCAAGGGC

	GAGGAGCTGTTCACCGGGGTGGTGCCCATCCTGGTCGAGCTGGACGGCGA<br>CGTAAACGGCCACAAGTTCAGCGTGTCCGGCGAGGGCGAGGGCGATGCC<br>ACCTACGGCAAGCTGACCCTGAAGTTCATCTGCACCACCGGCAAGCTGCC<br>CGTGCCCTGGCCCACCCTCGTGACCACCCTGACCTACGGCGTGCAGTGCT<br>TCAGCCGCTACCCCGACCACATGAAGCAGCACGACTTCTTCAAGTCCGCC<br>ATGCCCGAAGGCTACGTCCAGGAGCGCACCATCTTCTTCAAGGACGACGG<br>CAACTACAAGACCCGCGCCGAGGTGAAGTTCGAGGGCGACACCCTGGTG<br>AACCGCATCGAGCTGAAGGGCATCGACTTCAAGGAGGACGGCAACATCC<br>TGGGGCACAAGCTGGAGTACAACTACAACAGCCACAACGTCTATATCATG<br>GCCGACAAGCAGAAGAACGGCATCAAGGTGAACTTCAAGATCCGCCACA<br>ACATCGAGGACGGCAGCGTGCAGCTCGCCGACCACTACCAGCAGAACAC<br>CCCCATCGGCGACGGCCCCGTGCTGCTGCCCGACAACCACTACCTGAGCA<br>CCCAGTCCAAGCTGAGCAAAGACCCCAACGAGAAGCGCGATCACATGGT<br>CCTGCTGGAGTTCGTGACCGCCGCCGGGATCACTCTCGGCATGGACGAGC<br>TGTACAAGTAA
28 Å hairpin - mEGFP	ATGACCCGAACGGAAATCATTAGGGAGCTGGAGCGAAGTTTGCGCCTCCA<br>GCTGGTCCTTGCGATATTTCTGCTCGGACTTTTGATCGTACTTCTGTGGCT<br>GTTGCAGCAGCTGAAAGAACTGTTGCGGGAGCTTGAAAGGCTCCAACGG<br>GAGGGTAGCAGCGATGAGGACGTTCGGGAGCTGCTTAGGGAGATTAAGG<br>AGCTTGTGGAGAACATTGTTTATTTGGTCATTATTATCATGGTGTTGGTTC<br>TCGTAATAATAGCACTCACTGTAACTCAAAAGTATCTGGTGGAGGAACTT<br>AAACGGCAGGATGGCGGCGGATCCATGGTGAGCAAGGGCGAGGAGCTGT<br>TCACCGGGGTGGTGCCCATCCTGGTCGAGCTGGACGGCGACGTAAACGGC<br>CACAAGTTCAGCGTGTCCGGCGAGGGCGAGGGCGATGCCACCTACGGCA<br>AGCTGACCCTGAAGTTCATCTGCACCACCGGCAAGCTGCCCGTGCCCTGG<br>CCCACCCTCGTGACCACCCTGACCTACGGCGTGCAGTGCTTCAGCCGCTA<br>CCCCGACCACATGAAGCAGCACGACTTCTTCAAGTCCGCCATGCCCGAAG<br>GCTACGTCCAGGAGCGCACCATCTTCTTCAAGGACGACGGCAACTACAAG<br>ACCCGCGCCGAGGTGAAGTTCGAGGGCGACACCCTGGTGAACCGCATCG<br>AGCTGAAGGGCATCGACTTCAAGGAGGACGGCAACATCCTGGGGCACAA<br>GCTGGAGTACAACTACAACAGCCACAACGTCTATATCATGGCCGACAAGC<br>AGAAGAACGGCATCAAGGTGAACTTCAAGATCCGCCACAACATCGAGGA<br>CGGCAGCGTGCAGCTCGCCGACCACTACCAGCAGAACACCCCCATCGGC<br>GACGGCCCCGTGCTGCTGCCCGACAACCACTACCTGAGCACCCAGTCCAA<br>GCTGAGCAAAGACCCCAACGAGAAGCGCGATCACATGGTCCTGCTGGAG<br>TTCGTGACCGCCGCCGGGATCACTCTCGGCATGGACGAGCTGTACAAGTA<br>A
32 Å hairpin - mEGFP	ATGGGCTCGACCCGTACCGAAATCATTACCCGTCTGAGCTTCAGCCTGCT<br>GCTGCAGCTGGTTCTGGCGATTTTTCTGCTGGCGCTGCTGATCGTGCTGCT<br>GTGGCTGCTGCAGCAACTGAAGGAACTGCTGCGTGAGCTGGAACGTCTGC<br>AACGTGAGGGTAGCAGCGACGAAGATGTTCGTGAGCTGCTGCGTGAGATT<br>AAAGAACTGGTGGAGAACATCGTTTACCTGGTGATCATTATCATGGTGCT<br>GGTTCTGGTGATTATCGCGCTGGCGGTTCTGCAGATGTATCTGGTGCGTGA<br>ACTGAAGCGTCAAGACGGTAGCAGCGGATCCATGGTGAGCAAGGGCGAG<br>GAGCTGTTCACCGGGGTGGTGCCCATCCTGGTCGAGCTGGACGGCGACGT<br>AAACGGCCACAAGTTCAGCGTGTCCGGCGAGGGCGAGGGCGATGCCACC<br>TACGGCAAGCTGACCCTGAAGTTCATCTGCACCACCGGCAAGCTGCCCGT<br>GCCCTGGCCCACCCTCGTGACCACCCTGACCTACGGCGTGCAGTGCTTCA<br>GCCGCTACCCCGACCACATGAAGCAGCACGACTTCTTCAAGTCCGCCATG<br>CCCGAAGGCTACGTCCAGGAGCGCACCATCTTCTTCAAGGACGACGGCAA<br>CTACAAGACCCGCGCCGAGGTGAAGTTCGAGGGCGACACCCTGGTGAAC<br>CGCATCGAGCTGAAGGGCATCGACTTCAAGGAGGACGGCAACATCCTGG<br>GGCACAAGCTGGAGTACAACTACAACAGCCACAACGTCTATATCATGGCC<br>GACAAGCAGAAGAACGGCATCAAGGTGAACTTCAAGATCCGCCACAACA<br>TCGAGGACGGCAGCGTGCAGCTCGCCGACCACTACCAGCAGAACACCCC<br>CATCGGCGACGGCCCCGTGCTGCTGCCCGACAACCACTACCTGAGCACCC

	AGTCCAAGCTGAGCAAAGACCCCAACGAGAAGCGCGATCACATGGTCCT<br>GCTGGAGTTCGTGACCGCCGCCGGGATCACTCTCGGCATGGACGAGCTGT<br>ACAAGTAA
40 Å hairpin - mEGFP	ATGACTAGGACGGAGATTATAACTAGGCTCTCTTTTTCTCTTTTGTTGCAG<br>CTCGTGCTCGCTATATTTCTCCTTGCTCTTCTGATAGTCCTTCTTGTTCTGC<br>TTATCTATTTGAAGGAACTTCTCCGCGAGTTGGAGCGACTCCAGAGGGAG<br>GGGTCAAGCGACGAAGATGTACGAGAATTGTTGCGCGAAATTAAATGGTT<br>GGTAATTGTGATTGTGGCTCTCGTAATCATTATAATGGTCTTGGTATTGGT<br>AATCATCGCTCTTGCTGTGTTGCAAATGTACCTCGTTCGCGAACTGAAACG<br>GCAGGATGGCGGCGGATCCATGGTGAGCAAGGGCGAGGAGCTGTTCACC<br>GGGGTGGTGCCCATCCTGGTCGAGCTGGACGGCGACGTAAACGGCCACA<br>AGTTCAGCGTGTCCGGCGAGGGCGAGGGCGATGCCACCTACGGCAAGCT<br>GACCCTGAAGTTCATCTGCACCACCGGCAAGCTGCCCGTGCCCTGGCCCA<br>CCCTCGTGACCACCCTGACCTACGGCGTGCAGTGCTTCAGCCGCTACCCC<br>GACCACATGAAGCAGCACGACTTCTTCAAGTCCGCCATGCCCGAAGGCTA<br>CGTCCAGGAGCGCACCATCTTCTTCAAGGACGACGGCAACTACAAGACCC<br>GCGCCGAGGTGAAGTTCGAGGGCGACACCCTGGTGAACCGCATCGAGCT<br>GAAGGGCATCGACTTCAAGGAGGACGGCAACATCCTGGGGCACAAGCTG<br>GAGTACAACTACAACAGCCACAACGTCTATATCATGGCCGACAAGCAGA<br>AGAACGGCATCAAGGTGAACTTCAAGATCCGCCACAACATCGAGGACGG<br>CAGCGTGCAGCTCGCCGACCACTACCAGCAGAACACCCCCATCGGCGAC<br>GGCCCCGTGCTGCTGCCCGACAACCACTACCTGAGCACCCAGTCCAAGCT<br>GAGCAAAGACCCCAACGAGAAGCGCGATCACATGGTCCTGCTGGAGTTC<br>GTGACCGCCGCCGGGATCACTCTCGGCATGGACGAGCTGTACAAGTAA
50 Å hairpin - mEGFP	ATGGTGCTGTCTCATCATTTTGGCAAAGAATTCGCTAGCGCCACCATGAC<br>CCGCACGGAGATTATCACCAGGCTCAGTTTTTCCCTTTTGTTGCAACTTGT<br>CTTGGCAATTTTTTTGCTCGCACTGCTGATCGTACTCTTGGTGCTTTTGATA<br>GTTCTGATGATTCTCCTTATAGCGTTGGAATATCTTCAAAAAGAGGGATCT<br>TCAGATGAGGATGTGAAAGAACTCCTGGTGCTCATAATGATTTTGGTGAT<br>AGTGATTGTTGCCCTGGTAATTATAATCATGGTACTGGTCCTCGTTATAAT<br>CGCTCTGGCTGTGTTGCAGATGTACCTGGTTCGGGAACTCAAGCGACAAG<br>ACGGCGGCGGATCCATGGTGAGCAAGGGCGAGGAGCTGTTCACCGGGGT<br>GGTGCCCATCCTGGTCGAGCTGGACGGCGACGTAAACGGCCACAAGTTCA<br>GCGTGTCCGGCGAGGGCGAGGGCGATGCCACCTACGGCAAGCTGACCCT<br>GAAGTTCATCTGCACCACCGGCAAGCTGCCCGTGCCCTGGCCCACCCTCG<br>TGACCACCCTGACCTACGGCGTGCAGTGCTTCAGCCGCTACCCCGACCAC<br>ATGAAGCAGCACGACTTCTTCAAGTCCGCCATGCCCGAAGGCTACGTCCA<br>GGAGCGCACCATCTTCTTCAAGGACGACGGCAACTACAAGACCCGCGCC<br>GAGGTGAAGTTCGAGGGCGACACCCTGGTGAACCGCATCGAGCTGAAGG<br>GCATCGACTTCAAGGAGGACGGCAACATCCTGGGGCACAAGCTGGAGTA<br>CAACTACAACAGCCACAACGTCTATATCATGGCCGACAAGCAGAAGAAC<br>GGCATCAAGGTGAACTTCAAGATCCGCCACAACATCGAGGACGGCAGCG<br>TGCAGCTCGCCGACCACTACCAGCAGAACACCCCCATCGGCGACGGCCCC<br>GTGCTGCTGCCCGACAACCACTACCTGAGCACCCAGTCCAAGCTGAGCAA<br>AGACCCCAACGAGAAGCGCGATCACATGGTCCTGCTGGAGTTCGTGACCG<br>CCGCCGGGATCACTCTCGGCATGGACGAGCTGTACAAGTAA

50 Å hairpin – Malachite Green Aptamer	taatacgactcactatagggagaccacaacggtttccctctagaaataattttgtttaactttaagaaggagatatacaTAT GATGGTGCTGTCTCATCATTTTGGCAAAGAATTCGCTAGCGCCACCATGA CCCGCACGGAGATTATCACCAGGCTCAGTTTTTCCCTTTTGTTGCAACTTG TCTTGGCAATTTTTTTGCTCGCACTGCTGATCGTACTCTTGGTGCTTTTGAT AGTTCTGATGATTCTCCTTATAGCGTTGGAATATCTTCAAAAAGAGGGAT CTTCAGATGAGGATGTGAAAGAACTCCTGGTGCTCATAATGATTTTGGTG ATAGTGATTGTTGCCCTGGTAATTATAATCATGGTACTGGTCCTCGTTATA ATCGCTCTGGCTGTGTTGCAGATGTACCTGGTTCGGGAACTCAAGCGACA AGACGGCGGCGGATCCGACTATAAAGACGATGACGATAAATAAGtcgacGG GATCCCGACTGGCGAGAGCCAGGTAACGAATGGATCGGGTCGGCATGGC ATCTCCACCTCCTCGCGGTCCGACCTGGGCATCCGAAGGAGGACGTCGTC CACTCGGATGGCTAAGGGAGcggctgctaacaaagcccgaaaggaagctgagttggctgctgccacc gctgagcaataactagcataaccccttggggcctctaaacgggtcttgaggggttttttg
FLAG - 50 Å Hairpin-mEGFP	ATGGATTACAAGGATGACGACGATAAGcatATGGTGCTGTCTCATCATTTT GGCAAAGAATTCGCTAGCGCCACCATGACCCGCACGGAGATTATCACCA GGCTCAGTTTTTCCCTTTTGTTGCAACTTGTCTTGGCAATTTTTTTGCTCGC ACTGCTGATCGTACTCTTGGTGCTTTTGATAGTTCTGATGATTCTCCTTATA GCGTTGGAATATCTTCAAAAAGAGGGATCTTCAGATGAGGATGTGAAAG AACTCCTGGTGCTCATAATGATTTTGGTGATAGTGATTGTTGCCCTGGTAA TTATAATCATGGTACTGGTCCTCGTTATAATCGCTCTGGCTGTGTTGCAGA TGTACCTGGTTCGGGAACTCAAGCGACAAGACGGCGGCGGATCCATGGTG AGCAAGGGCGAGGAGCTGTTCACCGGGGTGGTGCCCATCCTGGTCGAGCT GGACGGCGACGTAAACGGCCACAAGTTCAGCGTGTCCGGCGAGGGCGAG GGCGATGCCACCTACGGCAAGCTGACCCTGAAGTTCATCTGCACCACCGG CAAGCTGCCCGTGCCCTGGCCCACCCTCGTGACCACCCTGACCTACGGCG TGCAGTGCTTCAGCCGCTACCCCGACCACATGAAGCAGCACGACTTCTTC AAGTCCGCCATGCCCGAAGGCTACGTCCAGGAGCGCACCATCTTCTTCAA GGACGACGGCAACTACAAGACCCGCGCCGAGGTGAAGTTCGAGGGCGAC ACCCTGGTGAACCGCATCGAGCTGAAGGGCATCGACTTCAAGGAGGACG GCAACATCCTGGGGCACAAGCTGGAGTACAACTACAACAGCCACAACGT CTATATCATGGCCGACAAGCAGAAGAACGGCATCAAGGTGAACTTCAAG ATCCGCCACAACATCGAGGACGGCAGCGTGCAGCTCGCCGACCACTACC AGCAGAACACCCCCATCGGCGACGGCCCCGTGCTGCTGCCCGACAACCAC TACCTGAGCACCCAGTCCAAGCTGAGCAAAGACCCCAACGAGAAGCGCG ATCACATGGTCCTGCTGGAGTTCGTGACCGCCGCCGGGATCACTCTCGGC ATGGACGAGCTGTACAAGTAA
20 Å hairpin - SNAP	ATGGGCTCGACTCGCAAGGAAATCATTGAGAAGTTAGAAAAGAGCCTGC GTCGCCAGAAGAAACTGGCACGTTTCCTTTTAATCTTACTTTTACTGTTGT TAGCTCTGCTGCTTGAATTATTGGAGCTTTTGCGCCGTCTGGAAGAGTTGC AGCGTCGCGGCTCAAGTGACGAAGAAGTCCATGAATTATTACGCCGTATC ATTGAGCTTGTGGAATATATTATCCTTCTGGTGTTGTTCATCATCGTACTT GTCCGCATCATCATCAAATTAGCAGAGCATCAACGCCGCTTGGTTGAGGA ACTGAAGAAGCAGGACGGTAGCAGCGGATCCATGGACAAAGATTGCGAA ATGAAACGTACCACCCTGGATAGCCCGCTGGGCAAACTGGAACTGAGCG GCTGCGAACAGGGCCTGCATGAAATTAAACTGCTGGGTAAAGGCACCAG CGCGGCCGATGCGGTTGAAGTTCCGGCCCCGGCCGCCGTGCTGGGTGGTC CGGAACCGCTGATGCAGGCGACCGCGTGGCTGAACGCGTATTTTCATCAG CCGGAAGCGATTGAAGAATTTCCGGTTCCGGCGCTGCATCATCCGGTGTT TCAGCAGGAGAGCTTTACCCGTCAGGTGCTGTGGAAACTGCTGAAAGTGG TTAAATTTGGCGAAGTGATTAGCTATCAGCAGCTGGCGGCCCTGGCGGGT AATCCGGCGGCCACCGCCGCCGTTAAAACCGCGCTGAGCGGTAACCCGGT GCCGATTCTGATTCCGTGCCATCGTGTGGTTAGCTCTAGCGGTGCGGTTGG CGGTTATGAAGGTGGTCTGGCGGTGAAAGAGTGGCTGCTGGCCCATGAAG GTCATCGTCTGGGTAAACCGGGTCTGGGATAA
24 Å hairpin - SNAP	ATGGGCACCAGGACAGAGATCATCAGGGAGCTTGAGCGATCCTTGCGAG AGCAGCGCGTGCTTGCTATTTTCCTCCTGGCGTTGCTCATCGTACTTCTCT

	GGCTGCTGCAACAACTTAAAGAATTGTTGCGCGAGCTGGAACGGCTGCAA<br>AGAGAAGGTTCATCCGACGAGGATGTAAGAGAATTGCTTAGAGAAATCA<br>AAGAACTTGTTGAGAACATTGTATATCTGGTAATAATAATCATGGTCCTC<br>GTCCTGGTAATCATAGCCCTGGCAAGAACGCAAAAATACCTTGTCGAAGA<br>GCTGAAGCGGCAGGATGGCGGCGGATCCATGGACAAAGATTGCGAAATG<br>AAACGTACCACCCTGGATAGCCCGCTGGGCAAACTGGAACTGAGCGGCT<br>GCGAACAGGGCCTGCATGAAATTAAACTGCTGGGTAAAGGCACCAGCGC<br>GGCCGATGCGGTTGAAGTTCCGGCCCCGGCCGCCGTGCTGGGTGGTCCGG<br>AACCGCTGATGCAGGCGACCGCGTGGCTGAACGCGTATTTTCATCAGCCG<br>GAAGCGATTGAAGAATTTCCGGTTCCGGCGCTGCATCATCCGGTGTTTCA<br>GCAGGAGAGCTTTACCCGTCAGGTGCTGTGGAAACTGCTGAAAGTGGTTA<br>AATTTGGCGAAGTGATTAGCTATCAGCAGCTGGCGGCCCTGGCGGGTAAT<br>CCGGCGGCCACCGCCGCCGTTAAAACCGCGCTGAGCGGTAACCCGGTGCC<br>GATTCTGATTCCGTGCCATCGTGTGGTTAGCTCTAGCGGTGCGGTTGGCGG<br>TTATGAAGGTGGTCTGGCGGTGAAAGAGTGGCTGCTGGCCCATGAAGGTC<br>ATCGTCTGGGTAAACCGGGTCTGGGATAA
40 Å hairpin - SNAP	ATGACTAGGACGGAGATTATAACTAGGCTCTCTTTTTCTCTTTTGTTGCAG<br>CTCGTGCTCGCTATATTTCTCCTTGCTCTTCTGATAGTCCTTCTTGTTCTGC<br>TTATCTATTTGAAGGAACTTCTCCGCGAGTTGGAGCGACTCCAGAGGGAG<br>GGGTCAAGCGACGAAGATGTACGAGAATTGTTGCGCGAAATTAAATGGTT<br>GGTAATTGTGATTGTGGCTCTCGTAATCATTATAATGGTCTTGGTATTGGT<br>AATCATCGCTCTTGCTGTGTTGCAAATGTACCTCGTTCGCGAACTGAAACG<br>GCAGGATGGCGGCGGATCCATGGACAAAGATTGCGAAATGAAACGTACC<br>ACCCTGGATAGCCCGCTGGGCAAACTGGAACTGAGCGGCTGCGAACAGG<br>GCCTGCATGAAATTAAACTGCTGGGTAAAGGCACCAGCGCGGCCGATGC<br>GGTTGAAGTTCCGGCCCCGGCCGCCGTGCTGGGTGGTCCGGAACCGCTGA<br>TGCAGGCGACCGCGTGGCTGAACGCGTATTTTCATCAGCCGGAAGCGATT<br>GAAGAATTTCCGGTTCCGGCGCTGCATCATCCGGTGTTTCAGCAGGAGAG<br>CTTTACCCGTCAGGTGCTGTGGAAACTGCTGAAAGTGGTTAAATTTGGCG<br>AAGTGATTAGCTATCAGCAGCTGGCGGCCCTGGCGGGTAATCCGGCGGCC<br>ACCGCCGCCGTTAAAACCGCGCTGAGCGGTAACCCGGTGCCGATTCTGAT<br>TCCGTGCCATCGTGTGGTTAGCTCTAGCGGTGCGGTTGGCGGTTATGAAG<br>GTGGTCTGGCGGTGAAAGAGTGGCTGCTGGCCCATGAAGGTCATCGTCTG<br>GGTAAACCGGGTCTGGGATAA
50 Å hairpin - SNAP	ATGGTGCTGTCTCATCATTTTGGCAAAGAATTCGCTAGCGCCACCATGAC<br>CCGCACGGAGATTATCACCAGGCTCAGTTTTTCCCTTTTGTTGCAACTTGT<br>CTTGGCAATTTTTTTGCTCGCACTGCTGATCGTACTCTTGGTGCTTTTGATA<br>GTTCTGATGATTCTCCTTATAGCGTTGGAATATCTTCAAAAAGAGGGATCT<br>TCAGATGAGGATGTGAAAGAACTCCTGGTGCTCATAATGATTTTGGTGAT<br>AGTGATTGTTGCCCTGGTAATTATAATCATGGTACTGGTCCTCGTTATAAT<br>CGCTCTGGCTGTGTTGCAGATGTACCTGGTTCGGGAACTCAAGCGACAAG<br>ACGGCGGCGGATCCATGGACAAAGATTGCGAAATGAAACGTACCACCCT<br>GGATAGCCCGCTGGGCAAACTGGAACTGAGCGGCTGCGAACAGGGCCTG<br>CATGAAATTAAACTGCTGGGTAAAGGCACCAGCGCGGCCGATGCGGTTG<br>AAGTTCCGGCCCCGGCCGCCGTGCTGGGTGGTCCGGAACCGCTGATGCAG<br>GCGACCGCGTGGCTGAACGCGTATTTTCATCAGCCGGAAGCGATTGAAGA<br>ATTTCCGGTTCCGGCGCTGCATCATCCGGTGTTTCAGCAGGAGAGCTTTAC<br>CCGTCAGGTGCTGTGGAAACTGCTGAAAGTGGTTAAATTTGGCGAAGTGA<br>TTAGCTATCAGCAGCTGGCGGCCCTGGCGGGTAATCCGGCGGCCACCGCC<br>GCCGTTAAAACCGCGCTGAGCGGTAACCCGGTGCCGATTCTGATTCCGTG<br>CCATCGTGTGGTTAGCTCTAGCGGTGCGGTTGGCGGTTATGAAGGTGGTC<br>TGGCGGTGAAAGAGTGGCTGCTGGCCCATGAAGGTCATCGTCTGGGTAAA<br>CCGGGTCTGGGATAA
TMH4C4 20 Å - FLAG	ATGAGTGCCGAGGAACTGCTGCGTCGTTCGCGCGAATATCTTAAGAAGGT<br>TGCTAAGTTTCAACTTGTGATCGCACTCGTATTCCTTATCCTGCTGGAAAT<br>CCTTTCGCGCCGTAGCGAGGAGCTGATCCGTGAATTAGAAGAGAAAGGC

	GCAGCCTCAGAGGCGGAGCTGGCCCGCATGAAACAACAACACATGACTG<br>CCTACCTGCAAGCCGCGTTAACCGCCTGGGAGATCATCAGCAAGAGCCTC<br>ATCGCCCTGTTATTACTCCAGCAGAATCAGCTCAATCTGGAACTTAACAC<br>GGATACAGACAAGAACGTAGCCGAGGAGTTACTTCGTCGTAGCCGTGAGT<br>ATCTTAAGAAGGTGGCGAAGAAGCAACTGGTTATTGCTTTTGTATTCCTC<br>ATCTTGCTCGAGATTTTAAGCCGCCGTAGTGAGGAGTTAATTCGTGAGTT<br>AGAGGAGAAGGGCGCGGCGTCGGAAGCCGAGTTGGCTCGCATGAAGCAA<br>CAACACATGACCGCCTATTTGCAGGCAGCGCTGACTGCCTGGGAGATCAT<br>TTCCAAATCTTTAATCGCGCTCCTGCTTCTGCAACAGAATCAACTGAATCT<br>CGAGCTCCGCCATGGCGGATCCGGGAGCGACTACAAAGACGATGACGAT<br>AAGTAA
TMH4C4 24 Å - FLAG	ATGTCCGCCGAGGAGTTACTGCGCCGTTCCCGCGAGTACTTAAAGAAGGT<br>CGCTAAAATTCAGCTGGTTATTGCTCTCGTCTTCTTGATCCTTCTCATCATT<br>TTGTCCCGCCGTAGCGAGGAACTCATCCGCGAACTCGAGGAGAAGGGCG<br>CCGCCAGCGAGGCCGAGTTGGCCCGCATGAAGCAACAACACATGACGGC<br>GTATTTGCAGGCCGCGTTGACTGCTTGGGAAATCATCTCAAAATCTGTTAT<br>TGCTCTGTTACTTTTGCAACAGAATCAATTAAATTTGGAGCTTAATACCGA<br>CACCGACAAGAATGTGGCTGAGGAGTTATTACGCCGCTCACGTGAGTATC<br>TGAAGAAGGTAGCAAAGATCCAGTTAGTTATCGCCTTCGTGTTCCTTATTC<br>TTCTGATCATTTTAAGCCGTCGCTCAGAGGAGCTGATTCGTGAGCTTGAA<br>GAAAAGGGAGCGGCTTCAGAAGCGGAACTGGCCCGTATGAAGCAACAGC<br>ACATGACGGCATATCTTCAGGCCGCGTTAACGGCTTGGGAGATTATTTCA<br>AAGAGTCGCATCGCATTGCTTCTGTTACAACAAAATCAGTTGAACCTGGA<br>GCTCCGTCACGGTGGATCCGGGTCAGACTATAAAGATGATGACGACAAGT<br>AA
TMH4C4 40 Å - FLAG	ATGTCAGCCGAGGAGTTGCTGCGTCGCTCTCGCAAGTACTTAATTTTGGTG<br>GCTTTGATTCAACTGGTGATCGCCTTCGTTTTCCTCATTCTTCTGATTCTTT<br>TGAGCTGGCTCTCATGGCTGTTAATTCGTGAGCTCGAGGAGAAAGGGGCA<br>GCGAGCGAGGCTGAGTTGGCGCGCATGAAGATTCAGATGATGACTGCCTA<br>TCTGCAGGCCGCGCTGACCGCCTGGGAGATCATTGTTAAAGCCGTTATTG<br>CCTTGCTCCTGCTCCGCCAAAATCAACTGAATCTTGAGCTCAACACTGAC<br>ACAGACAAGAATGTGGCAGAGGAGCTTCTCCGTCGTAGCCGCAAGTACTT<br>AATCATTGTGGCCTTAATTCAGTTGGTAATCGCATTCGTCTTTCTGATCCT<br>GTTAATCTTGCTTTCATGGAAGTCGTGGGAGCTGATTCGCGAGCTTGAAG<br>AGAAGGGCGCCGCCTCGGAAGCCGAACTCGCTCGTATGAAGATGCAAGT<br>GATGTTAGCCTATCTCCAAGCAGCTCTGACCGCTTGGGAAATTATTGCGA<br>AGTCAGTAATTGCGTTATTACTTCTCCTGCAAAACCAATTAAACCTGGAG<br>CTGCGCCACGGCGGATCCGGAAGTGACTACAAGGATGACGATGACAAGT<br>AA
TMH4C4 50 Å - FLAG	ATGTCTGCTGAGGAGTTGTTACGTTGGAGCCGCATTTATTTAGTCATCGTG<br>GCCTTAATCCAGTTGGTTATCGCTTTCGTATTCCTTATTTTGCTCATCTTGC<br>TGTCCTGGCTTTCATTAGTGCTCATCTGGGAGTTAGAGGAGAAGGGCGCT<br>GCAAGTGAGGCGGAGCTTGCGCGCATGATTCTCCAGGTCATGACGGCGTA<br>CTTGCAAGCAGCCTTAACTGCGTGGGAGATTATCGCAAAGGTCGTAATTG<br>CCCTGCTCCTGCTTGTGGTTAACCAGCTTAATCTCGAGCTGAACACGGAC<br>ACAGATAAGAACGTCGCCGAGGAGCTGTTACGCCGTTCCCTTTTGTATCT<br>GATCATGGTAGCCCTCATTCAATTAGTCATTGCATTCGTATTCCTTATCTT<br>GTTAATTCTCTTGAGCTGGATCTCGCTTCTGCTTATCTGGGAACTCGAGGA<br>AAAGGGCGCTGCGAGCGAAGCAGAGCTCGCCCGTATGGCGATCCAATTA<br>ATGATTGCTTATCTCCAAGCGGCCCTGACCGCATGGGAGATCATTGCAAA<br>GAGCGTCATCGCCTTGCTTCTTCTCATCTTAAATCAACTGAATCTTGAACT<br>GCGTCACGGAGGATCCGGTAGTGACTACAAGGACGACGACGACAAGTAA
20 Å Hairpin – FKBP - LgBit	ATGGGCTCGACTCGCAAGGAAATCATTGAGAAGTTAGAAAAGAGCCTGC<br>GTCGCCAGAAGAAACTGGCACGTTTCCTTTTAATCTTACTTTTACTGTTGT<br>TAGCTCTGCTGCTTGAATTATTGGAGCTTTTGCGCCGTCTGGAAGAGTTGC<br>AGCGTCGCGGCTCAAGTGACGAAGAAGTCCATGAATTATTACGCCGTATC

	ATTGAGCTTGTGGAATATATTATCCTTCTGGTGTTGTTCATCATCGTACTT<br>GTCCGCATCATCATCAAATTAGCAGAGCATCAACGCCGCTTGGTTGAGGA<br>ACTGAAGAAGCAGGACGGTAGCAGCGGATCCGCAAGTCCGGCAGCACCG<br>GCACCGGCATCACCAGCTGCACCAGCACCTAGTGCACCGGCAGGCGGTAT<br>TCTGTGGCATGAAATGTGGCACGAAGGTCTGGAAGAAGCAAGCCGTCTGT<br>ATTTTGGTGAACGTAATGTGAAAGGCATGTTTGAAGTTCTGGAACCGCTG<br>CATGCAATGATGGAACGTGGTCCGCAGACACTGAAAGAAACCAGCTTTA<br>ATCAGGCCTATGGTCGTGATCTGATGGAAGCACAAGAATGGTGTCGCAAA<br>TACATGAAAAGCGGTAACGTTAAAGATCTGCTGCAGGCATGGGATCTGTA<br>TTATCATGTTTTTCGTCGCATTAGCAAAGGTGGTAGCGGTGGTGGTGGTTC<br>TGGTGGTAGCAGCTCAGGTGGTGTTTTTACCCTGGAAGATTTTGTTGGTGA<br>TTGGGAACAGACCGCAGCATATAATCTGGATCAGGTGCTGGAACAAGGT<br>GGTGTGAGCAGCCTGCTGCAGAATCTGGCAGTTAGCGTTACCCCGATTCA<br>GCGTATTGTTCGTAGCGGTGAAAATGCCCTGAAAATTGATATTCATGTGA<br>TCATCCCGTATGAAGGTCTGAGCGCAGATCAGATGGCACAGATTGAAGAA<br>GTGTTCAAAGTTGTTTATCCGGTGGATGACCACCATTTTAAAGTTATTCTG<br>CCGTATGGCACCCTGGTTATTGATGGTGTGACCCCGAATATGCTGAATTAT<br>TTCGGTCGTCCTTATGAAGGTATTGCCGTTTTTGATGGCAAAAAAATCACC<br>GTTACCGGTACACTGTGGAACGGTAACAAAATTATCGATGAACGTCTGAT<br>TACACCGGATGGTAGCATGCTGTTTCGTGTTACCATTAACAGCTAA
50 Å Hairpin – FRB - SmBit	ATGGTGCTGTCTCATCATTTTGGCAAAGAATTCGCTAGCGCCACCATGAC<br>CCGCACGGAGATTATCACCAGGCTCAGTTTTTCCCTTTTGTTGCAACTTGT<br>CTTGGCAATTTTTTTGCTCGCACTGCTGATCGTACTCTTGGTGCTTTTGATA<br>GTTCTGATGATTCTCCTTATAGCGTTGGAATATCTTCAAAAAGAGGGATCT<br>TCAGATGAGGATGTGAAAGAACTCCTGGTGCTCATAATGATTTTGGTGAT<br>AGTGATTGTTGCCCTGGTAATTATAATCATGGTACTGGTCCTCGTTATAAT<br>CGCTCTGGCTGTGTTGCAGATGTACCTGGTTCGGGAACTCAAGCGACAAG<br>ACGGCGGCGGATCCGCAAGTCCGGCAGCACCGGCACCGGCATCACCAGC<br>TGCACCAGCACCTAGTGCACCGGCAGGCGGTGGTGTTCAGGTTGAAACCA<br>TTAGTCCTGGTGATGGTCGTACCTTTCCGAAACGTGGTCAGACCTGTGTTG<br>TTCATTACACCGGTATGCTGGAAGATGGCAAAAAATTCGATAGCAGCCGT<br>GATCGTAATAAGCCGTTTAAATTCATGCTGGGTAAACAAGAAGTTATTCG<br>CGGTTGGGAAGAGGGTGTTGCACAGATGAGCGTTGGTCAGCGTGCAAAA<br>CTGACCATTTCACCGGATTATGCCTATGGTGCAACCGGTCATCCGGGTATT<br>ATTCCGCCTCATGCAACCCTGGTTTTTGATGTTGAACTGCTGAAACTGGAA<br>GGTGGTAGCGGTGGTGGTGGTTCTGGTGGTAGCAGCTCAGGTGGTGTTAC<br>CGGTTATCGTCTGTTTGAAGAAATTCTGTAA

References:

1. Katira, S., Mandadapu, K. K., Vaikuntanathan, S., Smit, B. & Chandler, D. Pre-transition effects mediate forces of assembly between transmembrane proteins. *Elife* **5**, (2016).

2. Heberle, F. A. *et al.* Direct label-free imaging of nanodomains in biomimetic and biological membranes by cryogenic electron microscopy. *Proc Natl Acad Sci U S A* **117**, 19943–19952 (2020).

3. Nagle, J. F. & Tristram-Nagle, S. Structure of lipid bilayers. *Biochim Biophys Acta* **1469**, 159 (2000).
