# Supplementary material for: Hydrophobic mismatch drives self-organization of designer proteins into synthetic membranes
Source: Nat Commun. 2024 Apr 11;15:3162. doi: 10.1038/s41467-024-47163-1 (PMC11009411; doi:10.1038/s41467-024-47163-1)
Supplement: Supplementary file 2 — Reporting Summary [file 41467_2024_47163_MOESM2_ESM.pdf]

nature portfolio

Corresponding author(s): *Double-anonymous peer review submissions: write DAPR and your manuscript number here instead of author names.*

Last updated by author(s): *YYYY-MM-DD*

Reporting Summary

Nature Portfolio wishes to improve the reproducibility of the work that we publish. This form provides structure for consistency and transparency in reporting. For further information on Nature Portfolio policies, see our Editorial Policies and the Editorial Policy Checklist.

Statistics

For all statistical analyses, confirm that the following items are present in the figure legend, table legend, main text, or Methods section.

n/a	Confirmed	
☐	☒	The exact sample size (n) for each experimental group/condition, given as a discrete number and unit of measurement
☐	☒	A statement on whether measurements were taken from distinct samples or whether the same sample was measured repeatedly
☐	☒	The statistical test(s) used AND whether they are one- or two-sided<br>*Only common tests should be described solely by name; describe more complex techniques in the Methods section.*
☐	☒	A description of all covariates tested
☐	☒	A description of any assumptions or corrections, such as tests of normality and adjustment for multiple comparisons
☐	☒	A full description of the statistical parameters including central tendency (e.g. means) or other basic estimates (e.g. regression coefficient) AND variation (e.g. standard deviation) or associated estimates of uncertainty (e.g. confidence intervals)
☐	☒	For null hypothesis testing, the test statistic (e.g. F, t, r) with confidence intervals, effect sizes, degrees of freedom and P value noted<br>*Give P values as exact values whenever suitable.*
☒	☐	For Bayesian analysis, information on the choice of priors and Markov chain Monte Carlo settings
☒	☐	For hierarchical and complex designs, identification of the appropriate level for tests and full reporting of outcomes
☒	☐	Estimates of effect sizes (e.g. Cohen's d, Pearson's r), indicating how they were calculated

Our web collection on statistics for biologists contains articles on many of the points above.

Software and code

Policy information about availability of computer code

Data collection	All data were collected using stated instruments and associated commercially available software. No in-house algorithms or softwares were used to collect the data. Molecular Devices software was used to record plate reader data; Agilent Cary Eclipse Fluorescence Spectrophotometer software was used to record spectra for FRET studies.
Data analysis	FlowJo vl0.8 was used to analyze flow cytometry data. Excel (Microsoft), Prism v.9.0.0 (Graph Pad), Image-J v. 2.1.0 (NIH) for analyzing western blot and microscopy images.

For manuscripts utilizing custom algorithms or software that are central to the research but not yet described in published literature, software must be made available to editors and reviewers. We strongly encourage code deposition in a community repository (e.g. GitHub). See the Nature Portfolio guidelines for submitting code & software for further information.

Data

Policy information about availability of data

All manuscripts must include a data availability statement. This statement should provide the following information, where applicable:

- Accession codes, unique identifiers, or web links for publicly available datasets
- A description of any restrictions on data availability
- For clinical datasets or third party data, please ensure that the statement adheres to our policy

All data can be found in the manuscript and and supplementary files. DNA sequences encoding proteins and theirPlasmid maps, plasmid

descriptions, and plasmids used in each experiment can be found in the supplementary files. E, and experimental data,. including uncropped western blots, can be found in the Source Data.

Research involving human participants, their data, or biological material

Policy information about studies with human participants or human data. See also policy information about sex, gender (identity/presentation), and sexual orientation and race, ethnicity and racism.

Reporting on sex and gender	N/A
Reporting on race, ethnicity, or other socially relevant groupings	N/A
Population characteristics	N/A
Recruitment	N/A
Ethics oversight	N/A

Note that full information on the approval of the study protocol must also be provided in the manuscript.

Field-specific reporting

Please select the one below that is the best fit for your research. If you are not sure, read the appropriate sections before making your selection.

- [x] Life sciences
- [ ] Behavioural & social sciences
- [ ] Ecological, evolutionary & environmental sciences

For a reference copy of the document with all sections, see nature.com/documents/nr-reporting-summary-flat.pdf

Life sciences study design

All studies must disclose on these points even when the disclosure is negative.

Sample size	All spectroscopy and western blot experiments were performed 3 times (n=3). For all cell-free experiments, a replicate is defined as producing and preparing vesicles and cell-free reactions uniquely, three times. For flow cytometry, experiments were performed n=3, and 100,000 events were recorded for each replicate.
Data exclusions	None to report.
Replication	All attempts at replication were successful and biological triplicates of each sample were used to verify reproducibility
Randomization	No requirement for randomization to report. No animal or human participants were used in the study.
Blinding	Blinding was not relevant, as animal or human participants were not used in this study.

Reporting for specific materials, systems and methods

We require information from authors about some types of materials, experimental systems and methods used in many studies. Here, indicate whether each material, system or method listed is relevant to your study. If you are not sure if a list item applies to your research, read the appropriate section before selecting a response.

Materials & experimental systems

n/a	Involved in the study	
[ ]	[x]	Antibodies
[x]	[ ]	Eukaryotic cell lines
[x]	[ ]	Palaeontology and archaeology
[x]	[ ]	Animals and other organisms
[x]	[ ]	Clinical data
[x]	[ ]	Dual use research of concern
[x]	[ ]	Plants

Methods

n/a	Involved in the study	
[x]	[ ]	ChIP-seq
[ ]	[x]	Flow cytometry
[x]	[ ]	MRI-based neuroimaging

Antibodies

Antibodies used	1. HRP-anti-Mouse (CST 7076), 2.anti-GFP (Abcam), 3. Anti-DDDDK tag antibody (M2) (ab72469 4. DYKDDDDK Tag (D6W5B)

Validation	Antibody 1 was used as a secondary antibody for western blots and was validated by the manufacturer. Antibody 2 and 3 were used as primary antibodies for western blotting and were validated by Abcam. Antibody 4 was used for immunoprecipitation and flow cytometry experiments and was validated by CST. Antibody binding specificities and concentrations were validated by blotting against positive and negative protein controls and insuring proteins/antibodies yielded signal that was linear with concentration.

Plants

Seed stocks	N/A
Novel plant genotypes	N/A
Authentication	N/A

Flow Cytometry

Plots

Confirm that:

- [x] The axis labels state the marker and fluorochrome used (e.g. CD4-FITC).
- [x] The axis scales are clearly visible. Include numbers along axes only for bottom left plot of group (a 'group' is an analysis of identical markers).
- [ ] All plots are contour plots with outliers or pseudocolor plots.
- [x] A numerical value for number of cells or percentage (with statistics) is provided.

Methodology

Sample preparation	Vesicles and cell-free expressed proteins were prepared independently and each experiment was performed n=3.
Instrument	BD LSR Fortessa Special Order Research Product (Robert H. Lurie Cancer Center Flow Cytometry Core)
Software	Data was analyzed in FlowJo v10.8 and spectrally compensated. No custom code was used.
Cell population abundance	100,000 events were collected for all flow cytometry experiment. Percent abundance of each population is reported in the SI.
Gating strategy	Alexa Fluor 488 was excited with a 488 nm laser and captured with a 505 nm long pass filter and a 530/30 nm bandpass filter, Rhodamine was excited with a 552 nm laser and captured with a 582/15 nm bandpass filter, and Cy5.5 was excited with a 640 nm laser and captured with a 685 nm longpass filter and a 730/45 nm bandpass filter. Events on the cytometer were thresholded on the presence of either Rhodamine or CyS.5 detection to identify vesicles, and approximately 100,000 events were captured per reaction. Data was analyzed in FlowJo vl0.8 and spectrally compensated. Samples were gated using curly quad gating of Rhodamine versus Cy5.5 to isolate single-dye positive events and thus restrict analysis to only thin or thick membrane vesicles (Fig. 59). Curly quad gating (rather than quad gating) was necessary to account for photon counting and measurement error at high laser settings (50. Single lipid-dye positive events were then gated for Alexa Fluor 488 using samples containing vesicles but no Alexa Fluor 488. The percent of thin and thick vesicles identified as Alexa Fluor 488 positive and the mean fluorescent intensity (MFI) of each vesicle population was determined and analyzed. Vesicles that were prepared as above but that did not have a protein pore (i.e., the PURExpress reaction did not have pore-encoding DNA) was used to determine background. This background signal (e.g., percent positive vesicles or MFI) was subtracted as part of the data analysis process.

- [x] Tick this box to confirm that a figure exemplifying the gating strategy is provided in the Supplementary Information.
