# Supplementary material for: Hydrophobic mismatch drives self-organization of designer proteins into synthetic membranes
Source: Nat Commun. 2024 Apr 11;15:3162. doi: 10.1038/s41467-024-47163-1 (PMC11009411; doi:10.1038/s41467-024-47163-1)
Supplement: Supplementary file 3 — Description of Additional Supplementary Files [file 41467_2024_47163_MOESM3_ESM.pdf]

Title: Supplementary Movie 1.

Description: Video of 20 and 50 Å proteins in in phase separating membranes. The membrane is composed of 42.5 mol% DYPC/27.5 mol% DPPC/30 mol% Cholesterol. DYPC lipid (red) nucleates around the 20 Å hairpin protein (pink). The 50 Å protein (blue) is in contact with DPPC and cholesterol (grey) more often than DYPC. Further, proteins are apart from one another.
